# Natural variation at *FLM* splicing has pleiotropic effects modulating ecological strategies in *Arabidopsis thaliana*

**DOI:** 10.1038/s41467-020-17896-w

**Published:** 2020-08-18

**Authors:** Mathieu Hanemian, François Vasseur, Elodie Marchadier, Elodie Gilbault, Justine Bresson, Isabelle Gy, Cyrille Violle, Olivier Loudet

**Affiliations:** 1 Institut Jean-Pierre Bourgin, INRAE, AgroParisTech, Université Paris-Saclay, 78000 Versailles, France; 2 CEFE, Univ Montpellier, CNRS, EPHE, IRD, Univ Paul Valéry Montpellier 3, F-34090 Montpellier, France; 3 LEPSE, Univ Montpellier, INRAE, Institut Agro, F-34060 Montpellier, France; 4 Present Address: LIPM, INRAE, CNRS, Université de Toulouse, Castanet-Tolosan, France; 5 Present Address: GQE Le Moulon, INRAE, Univ. Paris-Sud, CNRS, AgroParisTech, Université Paris-Saclay, Gif-sur-Yvette, France

**Keywords:** Evolutionary genetics, Plant genetics, Natural variation in plants, Plant development

## Abstract

Investigating the evolution of complex phenotypes and the underlying molecular bases of their variation is critical to understand how organisms adapt to their environment. Applying classical quantitative genetics on a segregating population derived from a Can-0xCol-0 cross, we identify the MADS-box transcription factor FLOWERING LOCUS M (FLM) as a player of the phenotypic variation in plant growth and color. We show that allelic variation at *FLM* modulates plant growth strategy along the leaf economics spectrum, a trade-off between resource acquisition and resource conservation, observable across thousands of plant species. Functional differences at *FLM* rely on a single intronic substitution, disturbing transcript splicing and leading to the accumulation of non-functional *FLM* transcripts. Associations between this substitution and phenotypic and climatic data across Arabidopsis natural populations, show how noncoding genetic variation at a single gene might be adaptive through pleiotropic effects.

## Abstract (web summary)

*FLOWERING LOCUS M* (*FLM*) is known as a repressor of Arabidopsis flowering. Here, the authors show that a single intronic substitution of *FLM* modulates leaf color and plant growth strategy along the leaf economics spectrum, as well as plays a role in plant adaptation.

## Introduction

Evolution is a continuous and complex process requiring coordinated changes in many traits according to environmental selective pressure, in theory resulting in an optimal fitness in a given habitat at a given time point. Owing to the numerous traits that need to evolve coordinately, mathematical models predicted that complex organisms would evolve more slowly than simple organisms toward a fitness optimum when considering mutations of same effect size^1,2^. This was called the “cost of complexity” or the “cost of pleiotropy” since those models assumed that every gene is able to affect every trait.

Pleiotropy can have several meanings but overall states that one gene or variant controls several traits^3^. The nature of pleiotropy strongly depends on the traits measured (molecular, physiological, metabolic etc.) and the level of organization considered (from cell to population). Moreover, one needs to be cautious about the relevance of the observed pleiotropy according to the method used to assess it. For instance, studying knockout mutants may not reflect the diversity of mutational effects selected in nature. In addition, when classical quantitative genetics is used to study pleiotropy, a nontrivial point is to distinguish real pleiotropy from genetic linkage. Indeed, several independent quantitative trait loci (QTLs) in the same region may control different traits because mapping QTL in segregating populations has limited resolution with respect to the trait’s genetic architecture^4^. Finally, many related traits may be correlated just owing to a cause-and-effect relationship, in other words an indirect effect.

Reverse genetics and QTL mapping approaches in various organisms suggested that pleiotropy is relatively rare^5,6^. Moreover, a high degree of pleiotropy (i.e., the diversity of traits controlled by one variant/gene) is thought to limit the potential of adaptation because it would be more likely to affect negatively some functions while improving others (antagonistic pleiotropy), thus changing the optimal balance selected over the course of evolution. From this perspective, adaptation speed would rather depend on the degree and the modularity of pleiotropy (the combination of traits controlled by one variant/gene) than on the complexity of organisms^7,8^. Inspection of phenotypic data from yeast, nematode, and mice mutants revealed that an intermediate degree of pleiotropy seems the best trade-off to reach the highest rate of adaptation^6^. A recent study revealed that indeed, a combination of QTLs having an intermediate degree of pleiotropy, with QTLs affecting specifically one trait were able to promote a fast adaptation^9^.

In plants, phenotypic traits involved in the strategies for resource acquisition and use, called functional traits, have been widely investigated across species^10^. Several trait–environment relationships have been identified and are assumed to be representative of species adaptation to various environmental conditions. Ecologists have long recognized that functional traits are often correlated with each other, and that adaptation to new environments requires a simultaneous change in these traits due to resources limitation and biophysical constraints^11^. For instance, the rate of carbon fixation through photosynthesis is linked to the structure of the leaves, such as LMA and leaf dry matter content. Leaf structure and growth is also related to leaf chemical composition and its lifespan^12,13^. Together, these traits shape a trade-off between resource acquisition and resource conservation, bound by physiological constraints and observable across thousands of plant species. This trade-off has been called the “Worldwide Leaf Economics Spectrum”^12^, although it has also been observed between genotypes within species. For instance in the model species *Arabidopsis thaliana*, variations in the leaf economics spectrum (LES) are associated with life history traits’ variation between accessions, such as flowering time^14^.

Major developmental switches during plant growth cycle interact with plant physiology and fitness, among which the timing to flowering is expected to play a crucial adaptive role to ensure plant reproductive success. Some flowering time genes were shown to have pleiotropic functions during development^15^. Consistently, a previous study has shown tight relationships between flowering time and the vegetative growth dynamics in *A. thaliana*^16^. In *A. thaliana*, the molecular control of flowering time is orchestrated by a network of genes integrating several signals that converge towards a few regulators^17,18^. Among them, FLOWERING LOCUS C (FLC) is a MADS-box transcription factor known as a repressor of flowering^19^. Interestingly, FLC also plays a role in germination, another critical phase in plant development^20^ as well as vegetative development^21^. Furthermore, FLC was shown to bind to promoters of hundreds of genes involved in stress-related and hormonal pathways, activating or repressing their expression^22^. Natural variation of *FRIGIDA*, another major regulator of flowering time, modulates drought tolerance strategy through a pleiotropy exerted on several physiological traits^23^ and with different effects on plant water status if studied at a specific time point or integrated over the plant lifetime^24^. Indeed, the role of pleiotropy in drought adaptation was studied by assessing physiological traits that are correlated in nature such as water use efficiency (WUE) and flowering time^25^. Another example is that of *ERECTA* and its effect on functions like transpiration efficiency as well as inflorescence development^26^.

Examples of genes responsible for the natural variation of single traits are increasing (see for example^27–30^), however these studies are about a specific type of adaptation essentially having a simple genetic architecture in most studied populations. Studies aiming at the dissection of the genetic basis of phenotypic integration of several complex traits such as plant growth or fitness are scarcer and rarely reach the stage of the functional validation of the candidate genes and even less the identification of the causal natural variants^31,32^. Thus, dissecting the genetic architecture and identifying the molecular variants behind complex and/or correlated traits is crucial to fill this gap and to better understand how adaptation of organisms is constrained at the genetic level and the type of molecular variants selected (for instance regulatory versus protein coding).

To understand how phenotypic integration regulates plant adaptation to contrasted environments, we examine the genetic underpinnings of relationships between life history, leaf physiology, and climate. To do so, we seek to decompose complex traits into individual genetic and molecular components using a segregating population originating from a genetically divergent cross. We screen this population with a high-throughput phenotyping platform designed to allow great precision in measuring diverse traits, including highly integrative descriptors of plant growth. Growth is used as a model integrative trait to dissect relationships between traits’ variation and their adaptive potential. We identify the molecular basis of a QTL controlling both leaf vegetative growth and time to flowering in *A. thaliana*. Through a combination of quantitative genetics, molecular biology, ecophysiology, and population genetics, we show how modulating the transcriptional output of a gene can have a pleiotropic effect on multiple functions affecting life history and plant physiology, with potential impacts on ecological strategies. These results are exploited to distinguish pleiotropy from linkage in a concrete case, and to connect general theories about adaptation and pleiotropy.

## Results

### From several QTLs to a pleiotropic gene

**Fig. 1:**
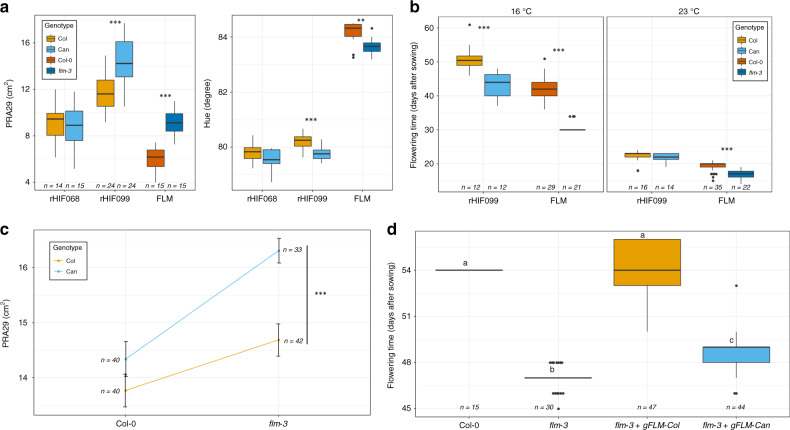
Natural variation at FLM is responsible for the QTLs observed at the end of chromosome 1. **a** Boxplots comparing PRA29 or Hue in alternate fixed alleles at the segregating region of the two most informative recombinant lines (rHIF068 is fixed at *FLM* but segregates elsewhere, and rHIF099 is segregating at *FLM*, see their exact genotype in Supplementary Data 4), and in *flm-3* versus its wild-type background Col-0. **b** Modulation of flowering time according to the growth conditions at 16 and 23 °C (in long days) in rHIF099 lines and *flm-3* versus Col-0. **c** Quantitative complementation assay assessing the effect on PRA29 of each of the possible genotypic combination between the Can or Col alleles at the QTL (in the rHIF background) and the wild-type or mutant (*flm-3*) alleles at *FLM* (in the Col-0 background). PRA29 was obtained using the *Phenoscope*. Error bars indicate the standard error of the mean. Significance is shown for the effect of the QTL allele in complementing *flm-3*. **d** Functional complementation based on flowering time of the *flm-3* mutant transformed with the genomic fragments of *FLM* (*gFLM*) from Col-0 or Can-0. Source data are provided as a Source Data file.

Using the *Phenoscope* phenotyping platform for *A. thaliana*^33^, we measured plant growth through projected rosette area 29 days after sowing (PRA29) during the vegetative phase of growth on 360 individuals of a recombinant inbred line (RIL) population derived from the cross between Can-0 and Col-0. We performed a QTL analysis to assess the genetic bases of variation in plant growth. In addition, several other image-derived traits such as leaf color, a surrogate for pigment concentration, were investigated^34^. QTLs for several of the measured traits co-localize at the bottom of chromosome 1 with mild effect on growth and large effect on leaf color (Supplementary Fig. 1A). We then followed a classical fine-mapping approach to identify the gene(s) underlying these trait variations. To this end, we selected the F7 RIL line 19RV337 with a residual heterozygous region covering the expected locus to build the heterogeneous inbred family (HIF) 19HV337 by selecting progenies homozygous for each of the parental allele at the candidate region. The phenotypic comparison of homozygous siblings confirmed that the allele originating from Can-0 (Can) was promoting PRA29 with respect to the allele originating from Col-0 (Col), and also influenced leaf color (Supplementary Fig. 1B). Phenotyping 12 selected recombinant HIF lines (rHIF) derived from 19HV337 confirmed the association of phenotypic variation with a candidate region containing ten genes (Supplementary Fig. 1C). Since the Can allele at this locus conferred both faster vegetative growth and earlier bolting with respect to the Col allele, we focused on the candidate gene coding for *FLOWERING LOCUS M* (*FLM*). We then used the null mutant *flm-3* in the Col-0 background. Compared to its wild-type (WT) control, this mutant displayed similar phenotypic effects on plant growth and color as rHIF099-Can compared to rHIF099-Col (our ultimate rHIF segregating at *FLM*), indicating that Can-0 could potentially harbor a hypofunctional *FLM* allele (Fig. 1a).

FLM is a MADS-box transcription factor inhibiting flowering at cool temperature and whose repressive action is alleviated when temperature increases moderately^35,36^. We observed that the differences in flowering time were stronger at cool temperature in rHIF099, similarly to the differences observed between *flm-3* and its WT counterpart (Fig. 1b). To further confirm *FLM* as the gene underlying the growth QTL, we performed a quantitative complementation assay^37^ relying on the comparison, in F1 hybrid plants, of the *Can* and *Col* alleles with respect to the *flm-3* allele. Hybrids were derived from crosses between rHIF099 (segregating at *FLM*) and *flm-3* or its WT background (Fig. 1c). Highly significant differences in plant growth (PRA29) were observed when *flm-3* was complemented by either of the QTL alleles (*p* = 2.638e−05). Although the significance of the interaction between the QTL allele and the mutant genotype was itself only marginal (*p* = 0.069), this overall indicates that the *Can* allele at *FLM* is less able to complement *flm-3* than the *Col* allele (Fig. 1c). Finally, we genetically transformed the *flm-3* mutant with a genomic fragment containing 3 kb of the promoter and the full *FLM* genomic region (including introns and the 3′UTR) from either *FLM-Can* or *FLM-Col*. *FLM-Col* fully complemented *flm-3* to WT level in terms of flowering time, unlike *FLM-Can*, confirming that this gene is hypofunctional in Can-0 (Fig. 1d). Altogether, these data show that allelic variation at *FLM* is underlying the plant growth and color QTLs segregating in Can-0 × Col-0 at the lower end of chromosome 1.

### Molecular bases of the natural variation at *FLM*

**Fig. 2:**
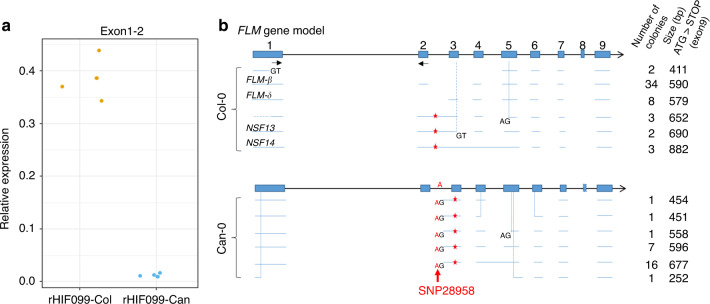
*FLM* transcripts characterization in Can-0 and Col-0. **a** Transcript accumulation was measured by qRT-PCR in rosette leaves grown on the Phenoscope from rHIF099 homozygous for the Can or the Col allele, using primers located in exon1 and exon2 of *FLM* (qFLM-F3 + qFLM-R3b), represented by black arrows in **b**. Each dot represents the mean of a technical duplicate from an individual plant. **b** PCR products of the *FLM* coding region of Col-0 and Can-0 (from the start to the stop codons) were used for subcloning and the colonies obtained were screened by PCR to estimate the size of many subcloned fragments. Plasmids of one or two colonies carrying a PCR product with a distinct band size were sent for Sanger sequencing. This provided the sequences illustrated here with the horizontal blue lines. An estimate of the frequency of each isoform sequenced is given according to the size of the PCR product obtained during the colony screening. The vertical dashed lines specify a discrepancy between the annotation and the isoform sequenced. New donor (GT) and acceptor (AG) splice site are depicted when present. Red stars represent premature stop codon. In Col-0, *FLM-β* as well as variants previously identified^38,39^ are mentioned (*FLM-δ*, *NSF13*, and *NSF14*). The candidate polymorphism responsible for the splicing shift observed in most of the *FLM-Can* transcripts is depicted in red (“SNP28958”). Source data underlying Fig. 2a are provided as a Source Data file.

Since de novo sequencing of PCR-amplified *FLM-Can* and *FLM-Col* alleles did not show any polymorphism within the coding sequence between these alleles (Supplementary Data 1 and Fig. 2), we assessed their expression with qRT-PCR. *FLM*-dependent thermosensitivity of flowering relies on a differential alternative splicing decreasing the production of the repressive isoform *FLM-β* when temperature increases^36,38–40^. Quantitative PCR showed that accumulation of exon2-containing transcripts (typical of *FLM-β* isoform) is strongly reduced in rHIF099-Can compared to rHIF099-Col (Fig. 2a).

Considering the complexity of the isoforms produced at the *FLM* gene according to a previous publication^39^, we thus set out to clone all the isoforms present in our samples. In Col-0, we found a majority of *FLM-β* isoform (34 out of 52 clones sequenced) as previously reported^39^ and discovered two isoforms including one which is potentially functional (Fig. 2b). In Can-0, we observed that most of the isoforms produced (26/27) contain an insertion of 17 bp before exon3 (Fig. 2b). Interestingly, a polymorphism changing a “GG” to an “AG” is located right before this additional stretch of DNA in Can-0 at the position 28,958,437. We deduced that this substitution in Can-0 (hereafter named “SNP28958”) is creating a consensus sequence of a splice acceptor site, which modifies *FLM* splicing and leads to the incorporation of these additional 17 nucleotides, producing a premature stop codon in the exon3. We showed that SNP28958 abolishes *FLM* function in term of flowering time, PRA29 and leaf color as the *flm-3* mutant was not complemented by an *FLM-Col* construct substituted with this SNP (Supplementary Fig. 3A). Moreover, we detected the *exon3*-containing isoform size shift in these transgenic plants (Supplementary Fig. 3B). Overall, it demonstrates that this single substitution is indeed creating a hot acceptor splice site generating nonfunctional *FLM* isoforms.

### Pleiotropic effects of *FLM* on leaf physiology

**Fig. 3:**
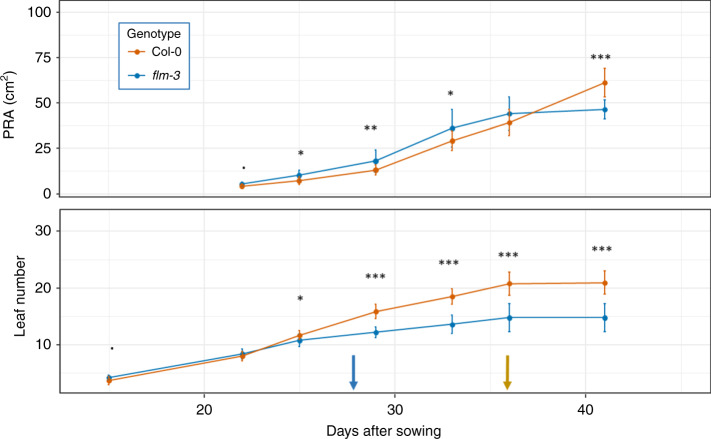
Relationships between vegetative growth and bolting time according to the *FLM* allele. Dynamics of projected rosette area (PRA) and leaf number followed until 41 days after sowing. Brown and blue arrow indicate the bolting time (floral stem 1 cm) in *flm-3* and Col-0 respectively. For each genotype, *n* = 12 individual plants. Data are presented as mean values ± s.d. Source data are provided as a  Source Data file.

We then wanted to describe how *FLM-Can* and *flm-3* alleles were affecting growth throughout the growth period under long day photoperiod, by comparison to the Col allele, using parallel PRA and leaf number measurements. As expected, we observed that both weak alleles promoted PRA in a first phase of vegetative growth (Fig. 3 and Supplementary Fig. 4). However, the growth of all lines slowed down and then stopped maximum 1 week after bolting, which means that the lines carrying a functional allele of *FLM* continued to grow over a longer period of time to reach the same (if not higher) final rosette sizes (Fig. 3 and Supplementary Fig. 4). Leaf number was rather similar in a first phase but lines carrying a functional *FLM* allele progressively developed more rosette leaves.

**Fig. 4:**
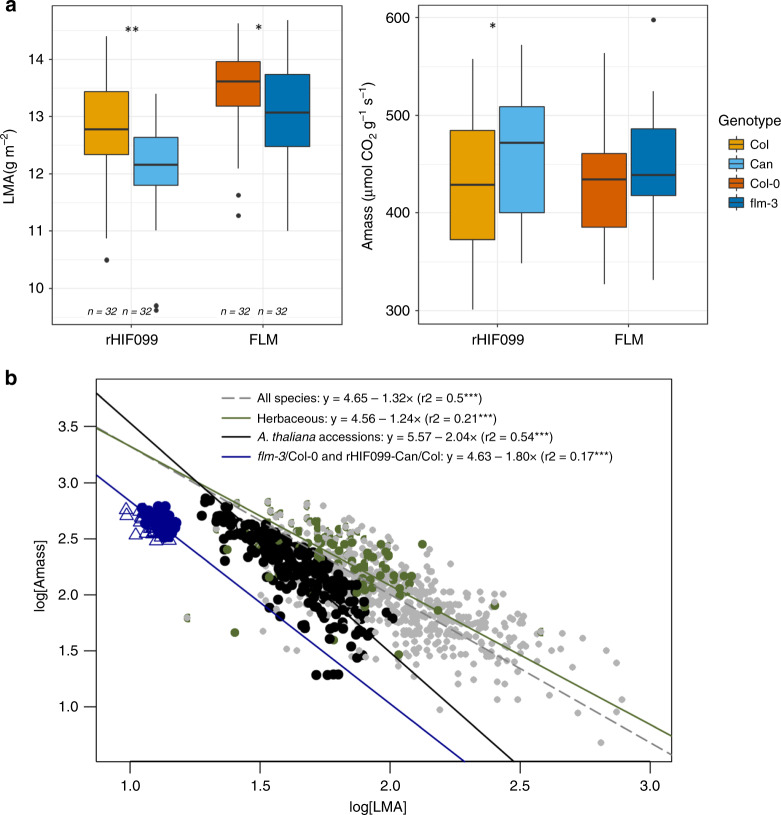
Allelic variation at *FLM* modulates important traits of the leaf economics spectrum. **a** Boxplots representing the difference in leaf dry mass per area (LMA, g m^−2^) and photosynthetic rate by unit of dry mass (*A*_mass_, nmol CO_2_ g^−1^ s^−1^) between the two allelic lines of rHIF099 as well as between *flm-3* and Col-0. Source data are provided in Supplementary Table 2. **b** Variations in leaf physiology associated with *FLM* variants compared to the worldwide leaf economics spectrum, i.e., the relationship between log_10_-transformed *A*_mass_ and LMA. Gray points represent all plant species from the GLOPNET dataset^12^ (*n* = 701). Green points represent only herbaceous species from the GLOPNET dataset (*n* = 124). Black circles represent natural accessions of *A. thaliana*^14^ (*n* = 340). Blue circles and triangles represent *flm-3*/Col-0 and the rHIF099-Can/Col plant groups measured in this study, respectively (*n* = 32 per genotype). Regressions have been estimated with standard major axis (SMA). *r*^2^: coefficients of correlation from SMA regressions. ***p < 0.001.

As *FLM* affected plant growth and color, we hypothesized that it might modulate leaf physiology through pleiotropic effects during vegetative growth. To test this hypothesis, we measured several traits related to leaf physiology and resource-use across a set of segregating lines and *flm* mutant. Leaf temperature measurements showed that leaves of rHIF099-Can and *flm-3* alleles were cooler compared to their respective controls, indicating a higher water flux through the plant (Supplementary Fig. 5). We also measured net photosynthetic rate per unit dry mass (*A*_mass_) and leaf dry mass per area (LMA), two core traits underlying variations in leaf physiology across and within species and illustrating physiological constraints observed in the plant kingdom. *flm-3* or *FLM-Can* were associated with significantly lower LMA when compared to their controls respectively in the Col-0 and rHIF099 genetic backgrounds, while *A*_mass_ was significantly higher only in the rHIF099 genetic background, although a similar trend was observed in the *flm-3*/Col-0 comparison (Fig. 4a). We also observed that *FLM* functional variation is associated with a negative correlation between these two traits, as observed across species and within *A. thaliana* natural accessions (Fig. 4b)^12,14^. Regression slopes were not significantly different (SMA test *p* > 0.05) when comparing our *FLM* dataset (rHIF099-Can/Col and *flm-*3/Col-0 together) with the variation observed across 340 natural *A. thaliana* (SMA slope in *FLM* = −1.80, 95% confidence interval = [−2.11;−1.54], *r*^2^ = 0.17; SMA slope in natural Arabidopsis accessions = −2.04, 95% confidence interval = [−2.16;−1.92], *r*^2^ = 0.54). By contrast, regression slopes were significantly different (SMA test *p* < 0.05) between our *FLM* dataset and interspecific measurements (SMA slopes in all species from the GLOPNET database = −1.32, 95% confidence interval = [−1.39;−1.25], *r*^2^ = 0.50; SMA slope in herbaceous species from the GLOPNET database = −1.24, 95% confidence interval = [−1.47;−1.04], *r*^2^ = 0.21), suggesting that although the LES represents an axis of physiological constraints between fast and slow growth strategies, this axis might vary slightly between species. Overall, these measurements showed that the leaf physiology associated with hypofunctional alleles at *FLM* was shifted along this LES axis toward more acquisitive strategies (i.e., a higher *A*_mass_ and a lower LMA). We estimated that the phenotypic consequences (along the LES axis) of hypofunctional alleles at *FLM* represents 2.3–3.8% (*flm-3* and *FLM-Can* alleles, respectively) of the range of variation observed among the 340 Arabidopsis accessions.

### *FLM* variation relevance in nature

**Fig. 5:**
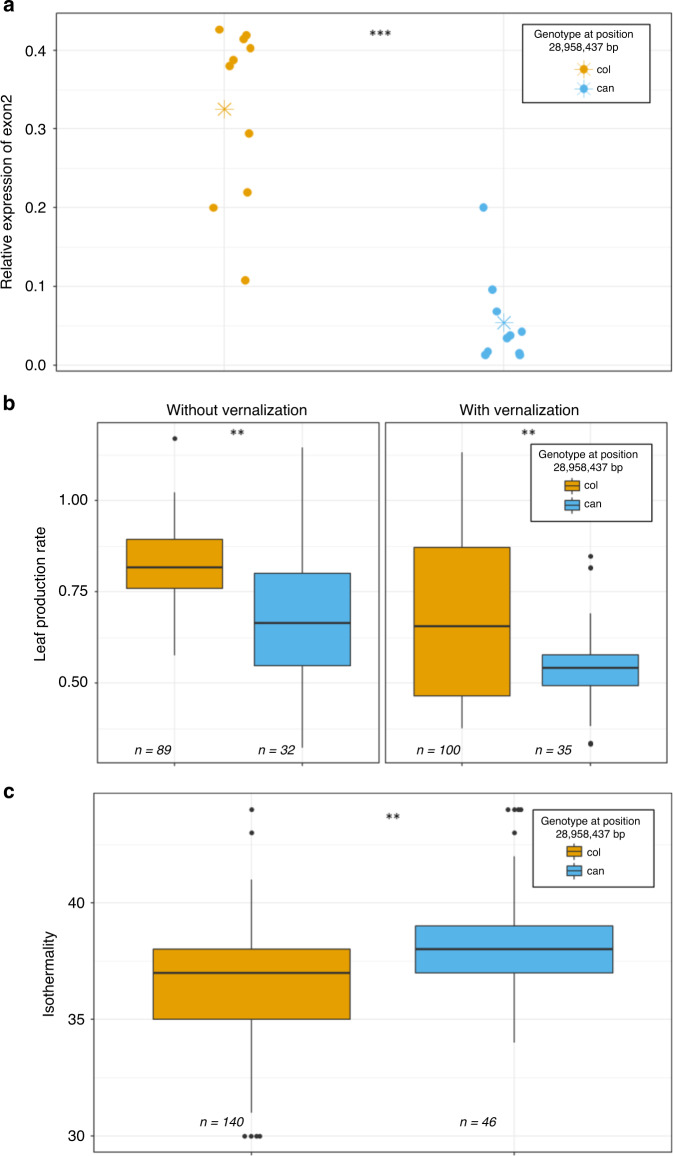
The variant at position 28,958,437 bp in Can-0 modulates *FLM* function and may play a role in adaptation. **a** Accumulation of exon2-containing isoforms (typical of *FLM-β*) measured by qRT-PCR using the primer pair qFLM-F3 + qFLM-R3b (see Fig. 2) in nine Arabidopsis strains from the Iberian Peninsula (IP) carrying either the *Can* (blue) or the *Col* (brown) genotype at position 28,958,437 bp. Each data point represents one IP strain. The asterisks are the mean expression of each variant. **b** Boxplots represent leaf production rate with or without vernalization in Iberian Peninsula strains categorized according to their genotype at position 28,958,437 bp. The data were obtained from a previous publication^42^ and are gathered in Supplementary Data 3. Significance of the differences was calculated according to a mixed-effect model using the genotype as a random factor and the kinship matrix as covariates (see Supplementary Table 1). **c** Boxplots represent isothermality in IP strains categorized according to their genotype at position 28,958,437 bp. Isothermality is calculated as follows: mean of monthly (max temp − min temp)/(max temperature of warmest month − min temperature of coldest month) × 100, and was obtained from http://www.worldclim.org/ and are gathered in Supplementary Data 3. Significance of the differences was calculated according to a mixed-effect model using the genotype as a random factor and the kinship matrix as covariates (see Supplementary Table 1). Source data underlying Fig. 5a are provided as a Source Data file.

To better understand the implications of the polymorphism identified, we screened all *A. thaliana* strains with data available for SNP28958^41^. Interestingly, out of the available 50 strains carrying this SNP, 45 come from the Iberian Peninsula (hereafter called IP strains), 2 from France, 2 from Italy and Can-0 from the Canary Islands (Supplementary Data 2). To evaluate the impact of SNP28958 on *FLM* expression in natural IP strains, two groups of nine IP strains carrying either the *Can* or the *Col* allele were selected for qRT-PCR experiments. A clear association between the allelic form and the accumulation level of exon2-containing transcripts was observed (Fig. 5a). Moreover, a shift in exon3-containing transcript size was only detected in the Can-like IP accessions indicating that this SNP most likely retain the same hypoactive molecular function as the *Can* allele (Supplementary Fig. 6).

We then wanted to know if the functional impact of SNP28958 on multiple traits could play a substantial role in IP strains adaptation to their habitat. We used a set of 186 IP strains for which we were able to gather geographic and phenotypic data from previous publications, including the 46 strains carrying SNP28958^29,42^ (Supplementary Data 3). We used this dataset to fit a general linear model corrected by a kinship matrix to avoid bias due to population structure (Supplementary Table 1). Consistent with our previous observations, we found significant associations between the *FLM* causal polymorphism and leaf production rate with or without vernalization (Fig. 5b).

Can-like IP strains do not cluster geographically and are scattered across Spain and Portugal (Supplementary Fig. 7). We used climatic variables (http://www.worldclim.org/) collected over 30 years to know whether the location of the Can-like IP strains was associated with particular environmental parameters. We found a significant association between the causal polymorphism and isothermality (*p* = 0.0017), which corresponds to the mean of the monthly temperature range during the day, divided by the maximal variation of temperature over the year (Fig. 5c). This suggests that the IP strains carrying the *FLM*-Can-like allele are rather located in areas where diurnal monthly temperatures are less homogeneous and could be interpreted as an adaptive response. This difference seems to be driven mainly by the monthly mean diurnal range for which a similar tendency is observed (*p* = 0.052).

### *FLM* allele evolution

**Fig. 6:**
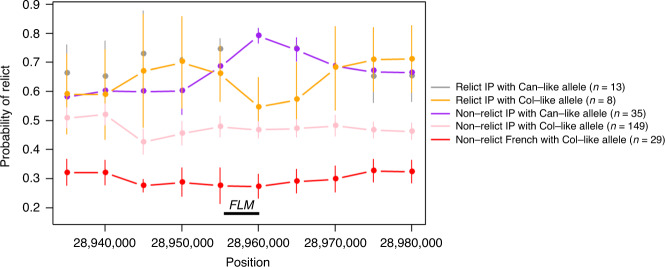
The variant at position 28,958,437 bp is associated with a relict origin. Curves show the average probability of haplotypes to be Iberian relicts around *FLM* using an analysis performed previously^43^. Each data point represents the mean over 10 kb sliding windows of each category sorted according to their relict origin (relict IP or non-relict IP) and their genotype at position 28,958,437 bp (Col-like or Can-like allele). Error bars represent confidence interval at 95%. Source data are provided as a Source Data file.

Pairwise genetic distances calculation among 1135 sequenced strains of *A. thaliana* recently showed that 26 of them, referred to as relicts, were highly divergent and are supposed to represent ancient lineages coming from ice-age refugia^41^. Among them, 21 strains form a group coming from the IP including 13 carrying the SNP28958 (Supplementary Data 3). This allele is thus enriched in the relict group (13/21 = 62%) compared to the overall IP (46/187 = 24.5%; *z* test: *p* = 0.00083). A complex pattern of genomic introgressions has recently been highlighted between relict and non-relict IP strains showing that their genome is rather a mosaic of the two groups^43^. We used this published dataset to estimate the probability of being relict around *FLM* in the IP strains according to the causal polymorphism. Interestingly, IP accessions diverge in their probability to be relict specifically around *FLM* according to SNP28958 and not their relict group (Fig. 6). It is also noteworthy that Can-0 belongs to an independent relict group^41^. Furthermore, the FLM-Can allele is also found in the 11 native Madeiran lines that are even more divergent than the relict groups identified in the first place, likely because they had less chance of admixture with non-relict strains^44^. Altogether, these data strongly suggest that the *FLM* allele from Can-0 has a relict origin, and at least a distinct admixed origin than the Col-0 allele.

## Discussion

Plant growth is a complex trait integrating variation from underlying features called functional traits^10^ such as net photosynthetic rate, nitrogen metabolism or WUE, themselves also affected by environmental cues throughout the plant life cycle. Plant growth variability is thus the output of the joint action of multiple genes at different levels of organization and is of potential adaptive significance. Likewise, studying this trait is of interest to better understand the genetic architecture and the degree of pleiotropy of traits under complex selective pressures. Here, we intended to map the genetic bases of multiple traits related to rosette growth in a RIL population using our phenotyping platform and focused on QTLs co-localized at the end of chromosome 1 having mild effects on plant growth. We found that this is the result of pleiotropic functions of the *FLM* gene highlighting that variation at *FLM* plays a much wider role than initially described. This makes an interesting study case to work on the relationships between trait complexity and pleiotropy in the light of adaptation.

Further phenotyping experiments showed that *FLM* is also controlling leaf temperature, photosynthetic rate per unit of leaf dry mass (*A*_mass_) and leaf mass per area (LMA). Interestingly, these are core traits of the LES, a universal trade-off between resource acquisition and resource conservation observed across thousands of plant species. The tight relationship between *A*_mass_ and LMA is supposed to reflect fundamental physiological constraints among plant species worldwide, shaping their diversification and local adaptation^11^. At one end of the spectrum are plants with resource-conservative strategies, characterized by long-lived, tough leaves with low nutrient concentration and low net photosynthetic rate. At the other end are plants with resource-acquisitive strategies, characterized by short-lived, flimsy leaves with high nutrient concentration and high net photosynthetic rate. The adaptive significance of the LES was recently shown using intraspecific data in *A. thaliana* suggesting that the LES is reflecting physiological constraints at the origin of growth variation and local adaptation at the leaf level^14^. From this perspective, we tested whether (1) phenotypic variability in our *A. thaliana* data is also bound by the physiological constraints underlined by LES, and (2) whether *FLM* might contribute to phenotypic variation within the LES, using comparisons between our dataset and the generic interspecific GLOPNET database^12^, as well as the global variation measured among *A. thaliana* natural accessions^14^. Our results revealed that *FLM* functional variation is constrained by a relationship between photosynthetic rate per unit of leaf dry mass and leaf mass per area, similarly to what is observed in the inter- and intraspecific datasets. However, slopes differed between *A. thaliana* (in our *FLM* dataset and among natural accessions) and interspecific measurements (in all or only herbaceous GLOPNET species). This suggests that the physiological constraints described by the LES might at least slightly differ between species, which can be explained by important evolutionary transitions and metabolic differences between plant species, such as deciduous–evergreen, herbaceous–woody, and annual–perennial. Second, our results show that functional variation at *FLM* is associated with coordinated changes in functional traits that modulate physiology along the resource acquisition/conservation axis of the LES. Thus, variation at a single flowering time gene has consequences on at least two core traits of the LES (*A*_mass_ and LMA), which is consistent with previous findings on flowering time genes in *A. thaliana*^31^. Strikingly, the impact of the *FLM* function in *A. thaliana* may represent about 3% of the worldwide variation in *A*_mass_ and LMA along the LES^12^. Two different scenarios may explain the range of phenotypic consequences caused by *FLM*. On the one hand, as physiological traits are highly interconnected^23^, *FLM* would modulate directly one of them e.g., photosynthetic rate of the plant, which in turn would indirectly change leaf mass per area, plant growth, and flowering time. This type of correlation was previously observed^31^ but the relationship(s) between leaf economics and flowering time is still unclear. On the other hand, *FLM* may activate or repress a set of target genes, selected through evolution owing to physiological constraints along the spectrum of leaf economics. Investigating the transcriptional network controlled by *FLM* might provide some answers as previously performed for *FLC*^22^. In parallel, it may be worth looking at the transcriptome response according to *FLM-β* expression level.

Although it is difficult to distinguish between a direct causality between traits due to *FLM* variation versus a synergistic pleiotropic effect along the LES axis, the associations between the *FLM* genotype and either growth strategy (leaf production rate) or the environment (isothermality) in natural populations would suggest an adaptive role for this variation. Interestingly, the knockout (*flm-3*) and the knockdown (Can) alleles of *FLM* used here displayed many features of the drought escape strategy, one of the strategies developed by the plants to overcome water shortage^45^. Indeed, these genotypes have a more rapid vegetative development, earlier flowering and higher photosynthetic rate, which would help them to complete their life cycle before a severe drought season^45^. To further validate this hypothesis, it would be interesting to investigate the adaptive value of *FLM* by combining water availability, temperature fluctuation, and neighbor-competition using our *Arabidopsis* transgenic lines differing only at SNP28958 or the Iberian natural accessions of each *FLM* group. Common garden experiments in locations differing in temperature fluctuations and precipitations could also be considered to quantify the adaptive effect of the *FLM* alleles in natural environments^32^.

FLM is a well-known transcription factor whose splicing variation in response to temperature change modulates the time to flowering^35,36^. *FLM-β* is the repressive variant of this gene, and its downregulation along increasing temperature triggers flowering^38,40,46,47^. The *FLM* coding sequences of Col-0 and Can-0 do not differ, but the typical *FLM-β* isoform is almost undetectable in Can-0 compared to Col-0. We identified a single nucleotide substitution in the 2nd intron of this gene (“SNP28958”), which is creating a seemingly hot acceptor splice site outcompeting other sites in Can-0 as well as in other related natural strains. This leads to the production of transcripts containing a premature stop codon. These aberrant variants may then be targeted by nonsense-mediated mRNA decay, as previously reported as a mechanism repressing *FLM* activity under higher temperature^39^. We have specifically validated the impact of the SNP28958 by testing its ability to change the functionality of *FLM-Col* in complementing the *flm-3* mutant phenotype.

Intronic polymorphisms were already shown to modulate *FLM-β* accumulation level in *Arabidopsis* natural strains and to correlate with flowering time^46,47^. This type of modification might be a way to modulate *FLM* function according to the thermosensitivity required in a given natural habitat, as the response to subtle changes in ambient temperature is crucial to set seeds at the appropriate time of the season. Loss-of-function mutations were proposed to play a substantial role in plant adaptation^48,49^. However, the selective value of such natural mutations was rarely demonstrated so far^50–52^. Even if it is challenging, this illustrates the value to identify causal genes as well as the causal polymorphisms involved in the natural variation of traits, as this type of subtle genetic variants are more difficult to reveal using large-scale approaches while they might play an important role in the course of evolution.

Many examples in human showed how natural variation in alternative splicing is altering gene functions, triggering numerous diseases^53^. In plants, examples are more scarce, yet alternative splicing is occurring in at least 30% of intron-containing genes in several plant species and is likely to play a role in adaptation^54–57^. For instance, sequence variation in introns of the *PYRROLINE-5-CARBOXYLATE SYNTHASE 1* gene was shown to disturb its proper splicing and consequently modifies proline accumulation, an important trait for drought and freezing tolerance^58^. In another example, a single polymorphism in *FLC* intron is changing splicing of the antisense transcript *COOLAIR* and then modulates *FLC* expression^59^. In both cases, an association between functional genetic variation and climatic variables was observed. Our work is contributing to this nascent topic since we found SNP28958 specifically in *Arabidopsis* strains of the IP. The categorization of the IP strains according to the genotype at this polymorphism allowed testing association with phenotypic and climatic variables. It is noteworthy that strains carrying SNP28958, i.e., the weak allele of *FLM*, which might thus be less responsive to ambient temperature change, are overall located in environments where the amplitude in temperature variation is more pronounced. Therefore, it is conceivable that this *FLM* allele is advantageous by desensitizing plants against fluctuating temperature to avoid the inappropriate triggering of flowering. Likewise, genetic variation affecting thermosensitivity of splicing in a key clock gene was proposed to drive thermal adaptation from equatorial to temperate climate in *Drosophila* species, supporting a similar hypothesis^60^.

The history of *Arabidopsis* migrations is regularly revisited as more data become available for population genetic studies^61,62^. IP is consistently mentioned as a location where highly diverged *Arabidopsis* lines (called relicts) are found in preserved and ancestral habitats^41,63,64^. We identified SNP28958 in Can-0 and we found this substitution in several IP strains, i.e., in 2 out of the 5 relict groups initially identified^41^. Interestingly, the frequency of SNP28958 is enriched 2.5 times among the relict IP strains compared to all IP strains. Furthermore, even among the non-relict IP strains carrying SNP28958, the genotype probability to be relict is specifically rising at the *FLM* locus, further supporting its relict origin. Clearly, the alleles of *FLM* described here have distinct admixed origins. Finally, we found SNP28958 in the 11 native Madeiran lines which are considered as some of the most divergent *Arabidopsis* strains so far^44^. *Arabidopsis* migrations were associated with climate disruptions such as temperature rise or precipitation fluctuation^61^. Regarding the pleiotropic functions of SNP28958 towards more acquisitive strategy, it is conceivable that this knockdown allele was adapted to ancestral habitats and was then outcompeted and wiped out by the *FLM-Col* allele during the last expansion of non-relict *Arabidopsis* in Europe^41,43^.

Since Darwin’s studies, the classical view in evolutionary biology is that adaptive evolution occurs through gradual steps and is therefore rather slow and highly polygenic^2,65^. However, an increasing number of studies showed that evolution of organisms can happen through more steep step for examples due to polyploidization events or to mutations in genes of large effect^66–68^. Under strong selective pressure, it seems logical that adaptation must be accelerated through large effect alleles for simple traits or by targeting pleiotropic genes if the given trait is more complex. While pleiotropy (and its role in adaptation) has been studied mainly theoretically for years, some empirical works relying on published mutant data or QTL analysis showed that pleiotropic genes are relatively rare in a range of organisms^5,6^. Recent works demonstrated that a combination of intermediate pleiotropic loci and loci having a specific function may constitute an optimal balance for a relatively fast adaptation and/or to manage complex traits, but the functional validation of candidate genes was not yet done^69,70^. Our study is in line with those works, and provide an important case study, as natural variation at *FLM*-coordinated traits is quantitatively associated with contrasted life strategy and leaf functions, and is associated with the environment. The determination of the causality relationships between traits with respect to variation at *FLM* still requires some work that will help us understand the patterns of selection for the different traits, and how this would be responsive in the face of climatic challenges.

## Methods

### Genetic material

A segregating population (named 19RV) derived from the cross between Can-0 (accession 163AV) and Col-0 (accession 186AV) is described and available from the Versailles Arabidopsis stock center^71^ (http://publiclines.versailles.inra.fr/). Description and associated data are gathered at http://publiclines.versailles.inra.fr/page/19. Three hundred fifty eight RILs were phenotyped across several biological replicates (independent experiments) on the Phenoscope^4^. HIF was fixed in a segregating progeny^4^ from the following RIL (19RV337) with a residual segregating region covering position 27,401,322 bp to 29,693,733 bp on chromosome 1 (see Supplementary Data 4). For fine mapping, 3360 progenies of heterozygous 19HV337 were genotypically screened to find recombinant individuals within the candidate region (rHIF). By interrogating the segregation of trait PRA29 in informative rHIFs, the candidate interval for the QTL was reduced to ~35 kb. The detailed genotype of 19HV337 and its rHIFs can be found in Supplementary Data 4. The mutant *flm-3* is a SALK T-DNA line kindly provided by Markus Schmid (Stock Center number N641971). Quantitative complementation assay was performed as described previously^37,72,73^ by crossing each rHIF allelic line to either the flm-3 mutant or its WT background (Col-0) and testing its phenotype directly in the F1 plants.

### Phenotyping and growth conditions

Initial RILs phenotyping, fine mapping, and mutant characterization were first obtained on the Phenoscope robots^33^ in well-watered conditions (60% soil water content saturation) as previously established^4^. The RIL set and the parental accessions have been phenotyped in two independent Phenoscope experiments, with one individual plant per RIL per experiment. The phenotypic values can be found in Supplementary Data 5. The growth room is set at an 8 h short days photoperiod (230 μmol m^−2^ s^−1^) with days at 21 °C/65%RH and nights at 17 °C/65% RH. Growth-related traits are extracted from daily images after segmentation (PRA = Projected Rosette Area = cumulative growth; RER = Rosette Expansion Rate = relative growth rate), as well as parameters describing the rosette color expressed in the HSV (Hue/Saturation/Value) scale.

Leaf temperature was measured on the Phenoscope with FLIR infrared cameras targeting the whole rosette as well as a wet paper control for room temperature. Normalized leaf temperature was calculated by subtracting room temperature to the average rosette temperature. The growth chambers used for the experiments carried out at 16 and 23 °C was set at 16 h long day photoperiod of 150 μmol m^−2^ s^−1^, 65% RH.

For the in vitro culture, seeds were surface-sterilized for 10 min in 70% EtOH, 0.1% TritonX-100, followed by one wash with 95% EtOH for another 10 min. Sterile seeds were then resuspended in a 0.1% agar solution and stratified in the dark at 4 °C for 3 days. Ten seeds per square Petri dishes (120 mm) containing typical Arabidopsis media^74^ were placed on one side and the Petri dishes set vertically. Plants were grown for 12 days in a culture room (21 °C, 16 h light/8 h dark cycle).

### QTL mapping

QTL mapping was performed using Multiple QTL Mapping algorithm implemented in the R/qtl package^75,76^. At first, genotypic missing data were augmented. Then, one marker every three markers were selected as cofactors and significant ones were selected through backward elimination (backward selection of cofactors). QTL was moved along the genome using these preselected markers as cofactors, except for the markers in the 25.0 cm window around the region of interest. QTL were identified based on the most informative model through maximum likelihood. According to permutation tests results, a conservative LOD threshold of 2.4 was applied to identify significant QTL.

### Measurement of physiological traits in greenhouse

*flm-3* knockout mutant and its WT (Col-0), as well as one recombinant HIF segregating for *FLM* (rHIF099) were grown in greenhouse at the Center d’Ecologie Fonctionnelle et Evolutive (CEFE, Montpellier, France) in fall 2017. Plants were grown in 32 replicates per genotype. Seeds were sown in 260 ml individual pots filled with a 1:1 (v:v) mixture of loamy soil and organic compost, and stratified at 4 °C for 3–10 days. At the emergence of the first two true leaves, plants were thinned to keep only one plant per pot. Pots were randomly distributed among four trays that were rotated every day in the greenhouse. All pots were watered twice a week. To reduce environmental heterogeneity in the greenhouse, walls were painted in white and a semitransparent curtain was installed below the glass roof. Additional light was provided to reach ca. 65 µmol m^−2^ s^−1^ PPFD. Photoperiod and temperature were kept constant at 12 h day length, and 18/16 °C day/night, respectively.

Gas exchanges were determined 28 days after the end of stratification, that is, just before harvest. The rate of CO_2_ assimilation per unit dry mass (*A*_mass_, nmolCO_2_ g^−1^ s^−1^) was measured using a whole-plant chamber designed for Arabidopsis (Li-Cor 6400-17, Li-Cor Inc., Lincoln, NE, USA) connected to a gas analyzer system (LI-6400XT; Li-Cor). *A*_mass_ was determined at steady state at 180 µmol m^−2^ s^−1^ PPFD, 20 °C) and at 390 ppm reference CO_2_. After gas exchange measurement, rosettes were cut, and then leaves were separated and scanned individually for measurements of leaf area (ImageJ 1.43C). Leaf blades were then oven dried at 65 °C for 72 h, and their dry mass (DM) was determined. Leaf dry mass per area (LMA, g m^−2^) was calculated as total blade DM divided by total blade area. The raw data can be found in Supplementary Table 2.

### Cloning procedures

*FLM* genomic fragments from Col-0 and Can-0 were amplified using Phusion high-fidelity Taq polymerase (Finnzymes, http://www.thermoscientificbio.com/finnzymes/) with a couple of primers including Gateway sites and flanking positions −2219 to +4737 relative to the *FLM* start codon of Col-0 annotated in TAIR10 (Supplementary Table 3). The pDONR207 entry vector containing the gFLM from Col-0 was mutagenized by PCR to substitute the nucleotide at the position +2759 by an “A”. Gateway-compatible fragments were cloned into the pDONR207 entry vector (Invitrogen) via BP recombination and subsequently transferred into the binary vector pFAST^77^ via a LR reaction following the Gateway cloning procedure (Invitrogen, www.invitrogen.com). After a verification done by Sanger sequencing, expression constructs were transformed into Agrobacterium tumefaciens strain C58C1. *flm-3* mutants were then transformed by floral dipping^78^ and transgenic plants were isolated using seed fluorescence. We selected four independent transgenic lines carrying the Col-0 and the Can-0 fragment of *FLM*, and two independent transgenic lines carrying the mutated version of *FLM*.

FLM transcripts variants of Col-0 and Can-0 were amplified using cDNAs obtained from pools of 3 rosettes grown at 23 °C with primers including start and stop codons using Phusion high-fidelity Taq polymerase. PCR products were cloned with the Zero Blunt TOPO PCR cloning kit (Invitrogen) following manufacturer specifications. Colony PCR was used to assess the size and the frequency of the different isoforms.

### Gene expression analysis

The RNeasy Plant Mini kit (Qiagen) was used for RNA extractions followed by a DNAse treatment (Fermentas). RT-PCR was performed on 500 ng of RNA using RevertAid H Minus reverse transcriptase (Fermentas) with oligo(dT) in 20 µL reactions. Then, 5 μl of tenfold diluted cDNAs was used for qRT-PCRs using a CFX96 real-time PCR machine (BioRad) with a SYBR solution (Eurogentec) using primers listed in Supplementary Table 3. Expression levels were normalized against the Arabidopsis *PP2A* gene (At1g13320). The qRT-PCR performed on the IP accessions were done on bulks of 12-day-old plantlets grown in vitro. All the other plants used for semiquantitative and quantitative RT-PCR were harvested from a Phenoscope experiment 29 days after sowing.

### Statistics and reproducibility

All boxplots are displayed following the same scheme: boxes show the interquartile range (i.e., the 1st and 3rd quartile) framing the median. The whiskers show the minimal and maximal values except if these values are higher than the interquartile range multiplied by 1.5. In this case, the whiskers show the interquartile range by 1.5 and the dots are outliers.

Significance of the difference in the boxplots were calculated with a two-sided Mann–Whitney–Wilcoxon test for the pairwise comparisons (“.”*p* < 0.1; “*”*p* < 0.05; “**”*p* < 0.01; “***”*p* < 0.001) in Figs. 1a–c, 2a, 3, 4a and Supplementary Figs. 1B, 4, 5 or with the Tukey HSD test without adjustment for multiple comparisons, the different letters representing groups at *p* < 0.01 in Fig. 1d and Supplementary Fig. 3A.

Linear regressions between *A*_mass_ and LMA were examined with standard major axis (SMA), using the package *smatr* in R^79^. Physiological traits (i.e., LMA and *A*_mass_) across Arabidopsis accessions were obtained from^14^, while physiological traits from interspecific data were obtained from the GLOPNET database^12^. *FLM* datasets (in the Col-0 as well as in the rHIF099 background) were orthogonally projected on the intraspecific Arabidopsis LES regression to estimate the contribution of variation at *FLM* to the species-wide LES range (*flm-3*/WT or FLM-Can/FLM-Col LES range expressed in percentage of the 95%-confidence interval of the 340 accessions LES range). All analyses were performed in R 3.2.3 (Team RC 2014).

Trait–climate relationships were modeled with linear mixed-effect models, using the *lmekin* function from the *coxme* package in R. Relatedness matrix between the IP accessions was included as covariate in the model. Relatedness matrix was calculated from whole-genome sequences^41^ with the function--*make-rel* from PLINK^80^.

### Reporting summary

Further information on research design is available in the Nature Research Reporting Summary linked to this article.

## Supplementary information

Supplementary Information

Peer Review

Reporting Summary

Description of Additional Supplementary Files

Supplementary_Data_1

Supplementary_Data_2

Supplementary_Data_3

Supplementary_Data_4

Supplementary_Data_5

## Data Availability

Data supporting the findings of this work are available within the paper and its Supplementary Information files. A reporting summary for this Article is available as a Supplementary Information file. The datasets generated and analyzed during the current study are available from the corresponding author upon request. The data used for Fig. 4a are supplied as Supplementary Table 2. The data used for Fig. 5b, c are supplied as Supplementary Data 3. The data used for Supplementary Fig. 1A are supplied as Supplementary Data 5. The data used for Fig. 4b can be found in Wright et al.^12^ [https://static-content.springer.com/esm/art%3A10.1038%2Fnature02403/MediaObjects/41586_2004_BFnature02403_MOESM2_ESM.xls] for the “all species” and “Herbaceous” datasets, and in INRAE data repository [10.15454/B3W0OS] for the “*A. thaliana* accessions” dataset. Source data are provided with this paper.

## Source data

Source Data
