# Supplementary material for: Natural variation at FLM splicing has pleiotropic effects modulating ecological strategies in Arabidopsis thaliana
Source: Nat Commun. 2020 Aug 18;11:4140. doi: 10.1038/s41467-020-17896-w (PMC7435183; doi:10.1038/s41467-020-17896-w)
Supplement: Supplementary file 1 — Supplementary Information [file 41467_2020_17896_MOESM1_ESM.pdf]

Natural variation at *FLM* splicing has pleiotropic effects modulating ecological strategies in *Arabidopsis thaliana*

Hanemian *et al.*

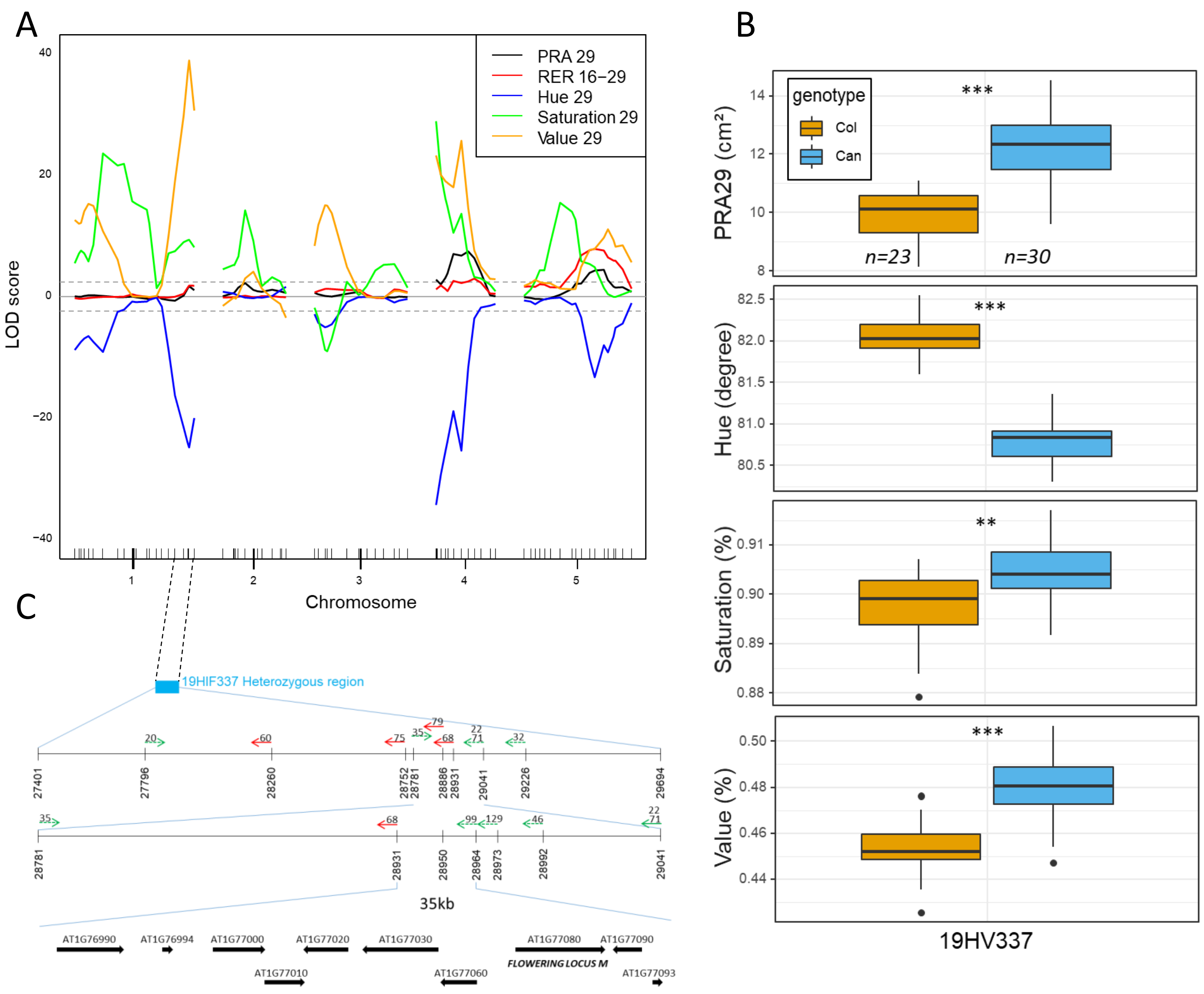

Supplementary Figure 1. QTL mapping and fine-mapping of the QTLs located at the end of chromosome 1.

A. QTLs from the Can-0 x Col-0 RIL set for 5 traits: Projected Rosette Area 29 days after sowing (PRA29), Relative Expansion Rate from 16 to 29 days after sowing (RER16-29) and average rosette colour expressed in the HSV (Hue, Saturation, Value) scale. Positive or negative LOD score values indicate that the *Can* allele or the *Col* allele increases trait value, respectively. Significance threshold (LOD=2.4) is shown as dotted lines. Raw data are provided in Supplementary Data 5.

B. Validation of the QTLs located at the end of chromosome 1 using the heterogeneous inbred family 19HV337 segregating for the region highlighted with the blue rectangle in panel C. The box plots for PRA29, Hue, Saturation and Value show the distribution of the phenotypic data and significance of the genotype effect.

C. Analysis of PRA29 segregation in series of recombinants issued from 19HV337[Het] reduced the QTL candidate interval to 35kb. Each arrow is located at the recombination breakpoint of a recombinant HIF line (rHIF). The back of the arrow indicates the fixed (homozygous) region whereas the head points toward the remaining segregating region. Dashed green and filled red colours indicate whether PRA29 is segregating or not in this rHIF, respectively. The thicker black arrows at the bottom represent the candidate genes present in the final 35kb-interval of interest.

Source data underlying Supplementary Figure 1B are provided as a Source Data file.

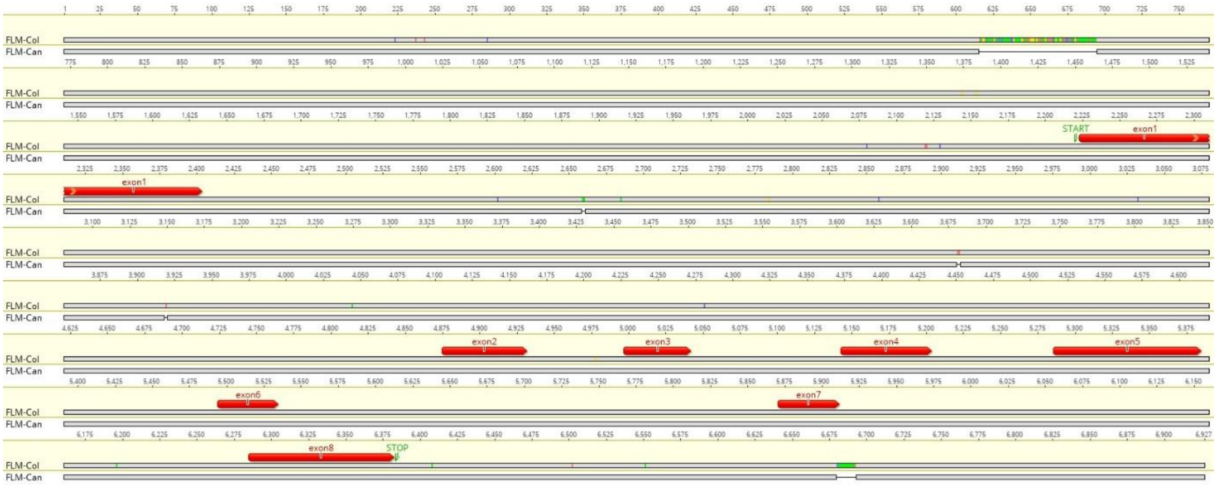

Supplementary Figure 2. Alignment of the genomic sequences of the *FLM* genes from Col-0 and Can-0.

The alignment was performed with the Geneious software using the sequence available in Supplementary Data 1. Polymorphisms are highlighted in the top row in which *FLM-Col* is used as a reference. Exons are depicted in red, START and STOP codons in green.

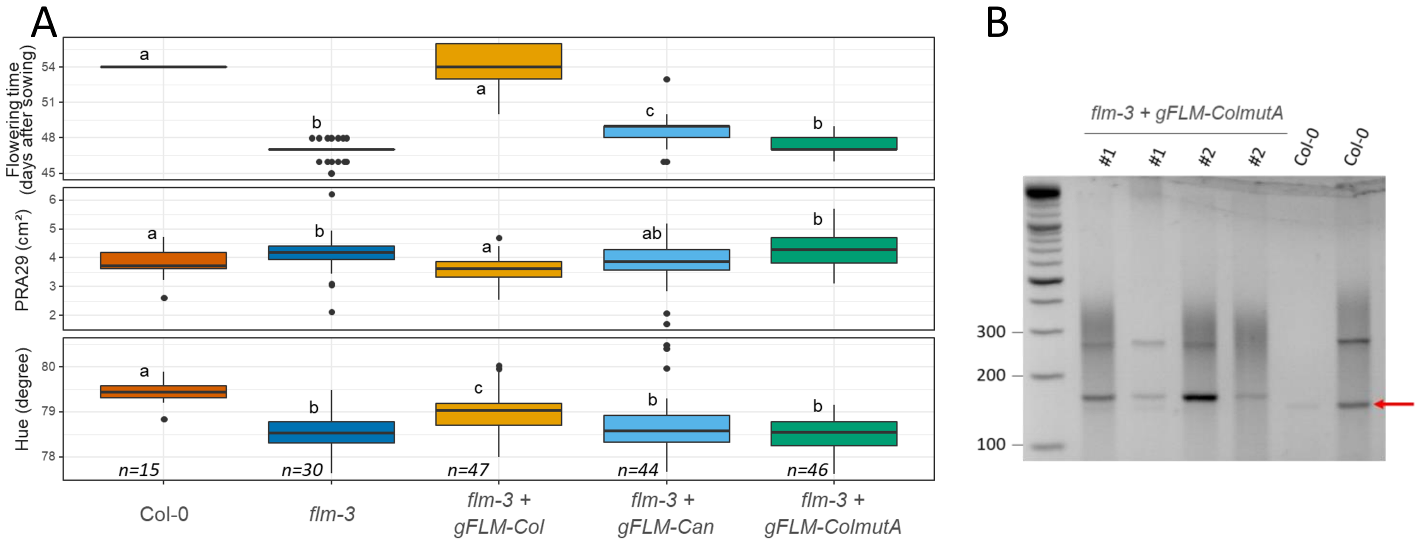

Supplementary Figure 3. Functional complementation of *flm-3.*

A. Different genomic fragments (promoter to 3'UTR) of *FLM* were used to transform the *flm-3* mutant for functional complementation assays: the fragments of Can-0 (gFLM-Can), Col-0 (gFLM-Col) and Col-0 substituted with SNP28958 (gFLM-ColmutA).The data presented here as boxplots for flowering time, PRA29 and Hue were obtained from T2 plants (4 independent lines for gFLM-Can and gFLM-Col, 2 independent lines for gFLM-ColmutA).

B. Characterization of *exon3*-containing isoforms by PCR using the primer pair qFLM-F3+qFLM-R3d which is spanning 'SNP28958', in 2 individuals of each gFLM-ColmutA T2 lines and 2 Col-0 plants as a control. The red arrow indicates the expected size in Col-0. The molecular-weight size marker on the side of the gel is expressed in bp. We repeated this experiment at least twice for each line and we obtained the same results.
Source data are provided as a Source Data file.

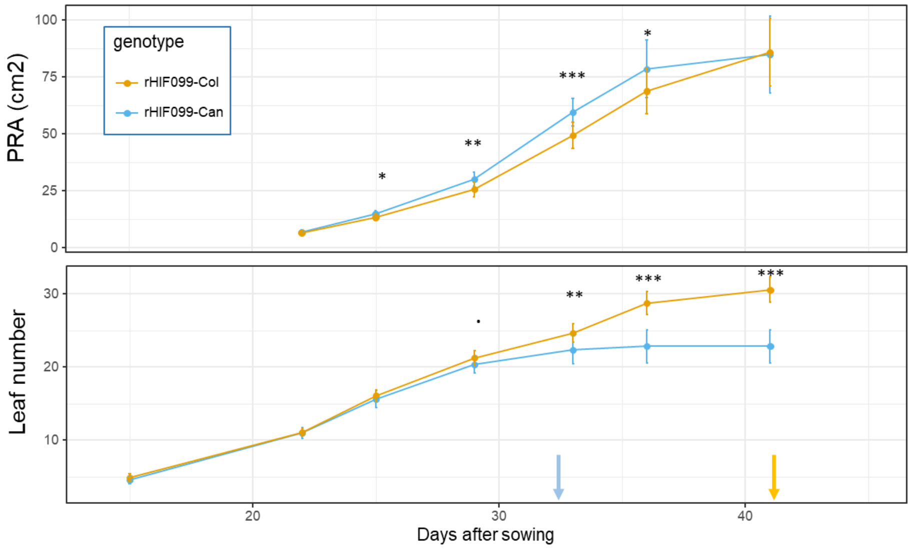

Supplementary Figure 4. Relationships between vegetative growth and bolting time according to the *FLM* allele.

Dynamics of projected rosette area (PRA) and leaf number followed until 41 days after sowing. Brown and blue arrow indicate the bolting time (floral stem 1cm) in rHIF099-Can and rHIF099-Col respectively. For each genotype, n=12 individual plants. Data are presented as mean values +/- s.d.. Source data are provided as a Source Data file.

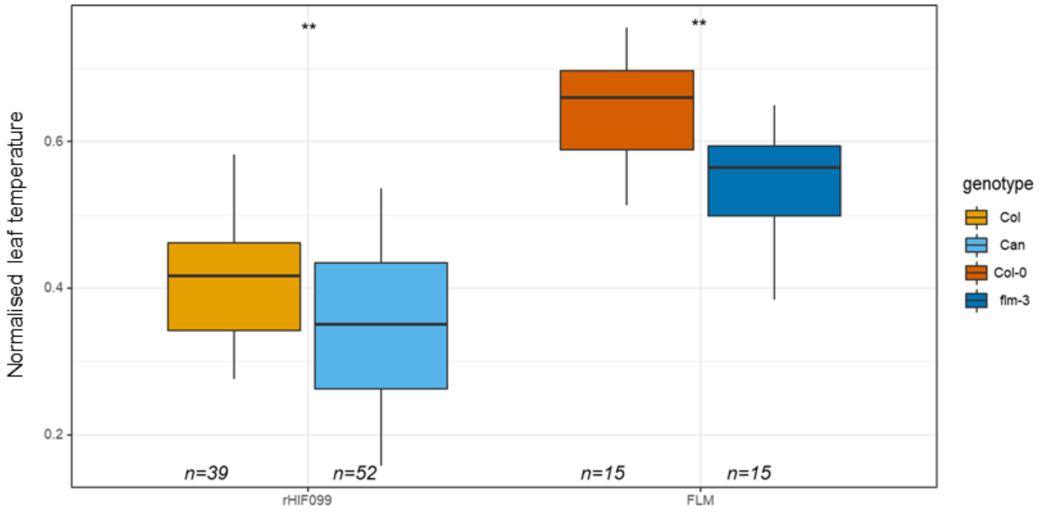

Supplementary Figure 5. Leaf temperature is affected by the allele at *FLM.*

The data points presented as boxplots were obtained by normalizing the mean of rosette temperatures with the room temperature. Source data are provided as a Source Data file.

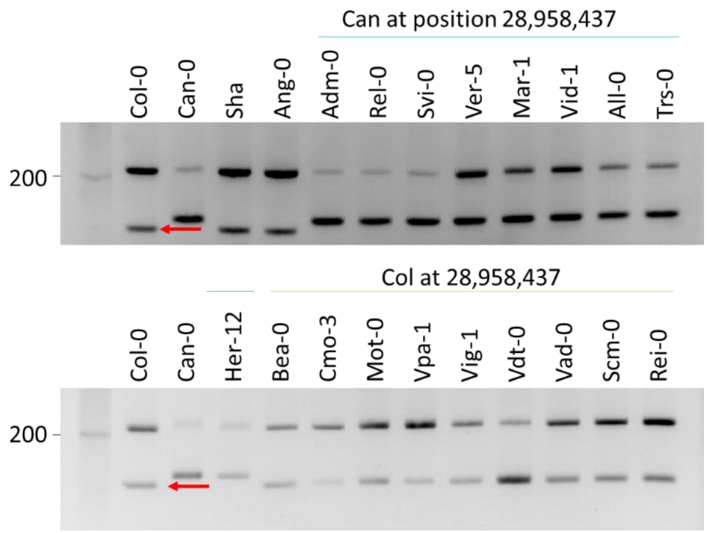

Supplementary Figure 6. The variant at position 28,958,437bp changes splicing in natural IP strains.

Characterization of the splicing shift at *exon3* by PCR using the primer pair qFLM-F3+qFLM-R3d on cDNA obtained from a bulk of 12 days-old plantlets grown in vitro from Col-0, Can-0, Sha, Ang-0 and 9 IP strains carrying either the *Can* (blue) or the *Col* (brown) allele at position 28,958,437bp. The red arrows indicate the expected size in Col-0. The molecular-weight size marker on the side of the gel is expressed in bp. We repeated this experiment at least twice for each genotype and we obtained the same results. Source data are provided as a Source Data file.

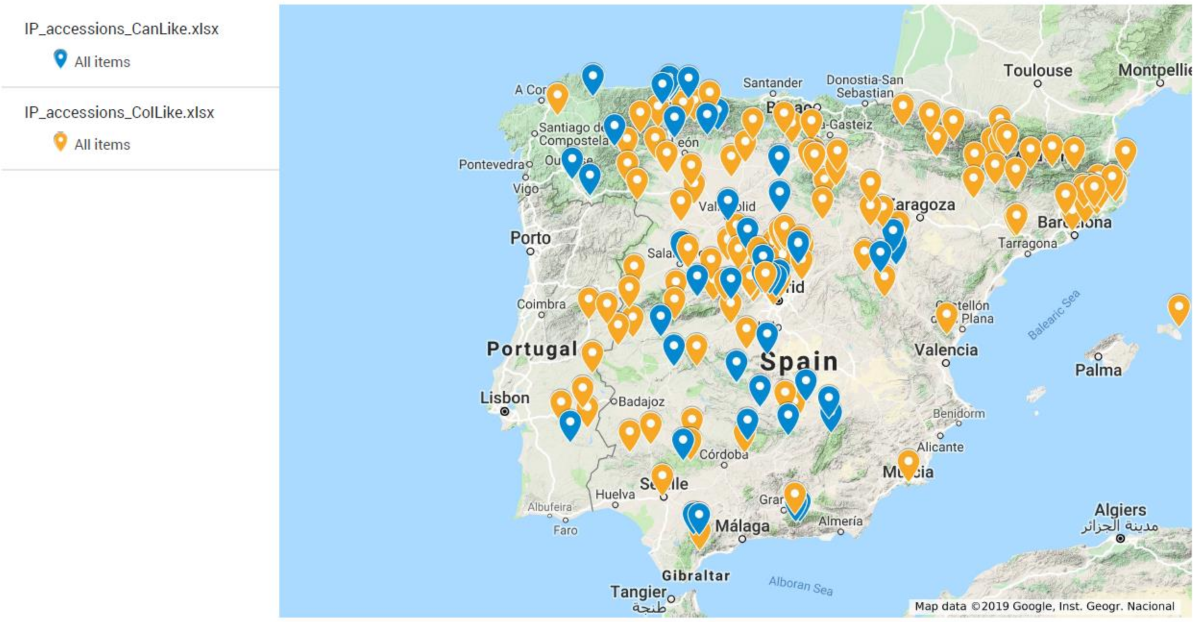

Supplementary Figure 7. Geographical origin of the IP strains.

Map obtained from Googlemap showing the location of the IP strains used in this study for the association analyses. Location points are respectively blue or brown according to the *Can* or *Col* genotype at position 28,958,437bp on chromosome 1.

Supplementary Table 1. Associations between the polymorphism at the position 28,958,437 and phenotypic or climatic variables gathered in Supplementary Data 3.

Variable	*p*-value of FLM Haplotype	Significance level
OVR	0,43	
LN	0,14	
FT2011	0,25	
LPR	0,0071	**
VLN	0,051	.
VFT	0,1	
VLPR	0,0091	**
SLN	0,99	
SFT	0,64	
Latitude	0,69	
Longitude	0,053	.
Altitude	0,86	
bio1	0,53	
bio2	0,052	.
bio3	0,0017	**
bio4	0,62	
bio5	0,39	
bio6	0,76	
bio7	0,48	
bio8	0,71	
bio9	0,79	
bio10	0,63	
bio11	0,45	
bio12	0,69	
bio13	0,91	
bio14	0,35	
bio15	0,18	
bio16	0,91	
bio17	0,26	
bio18	0,27	
bio19	0,97	

Model: Y ~ Haplotype + (1|Ecotype.ID), varlist=kinship_matrix

Signifiance codes: 0 '***' 0.001 '**' 0.01 '*' 0.05 '.' 0.1 ' ' 1

Supplementary Table 2. Measurements of physiological traits.

genotype	line	date_t0growth	date_PN	DM_Leaves (g)	Leaves_area_scan (cm^2)	SLA (m^2.kg-1)	Photo_DM_Leaves (µmolC02.g-1.s-1)	LMA (g.m-2)	Amass (nmolC02.g-1.s-1)
Col-0	Col_P1	9/22/2017	10/20/2017	0.0435	32.88	75.59	0.38223615	13.2292631300437	382.23615
Col-0	Col_P1	9/25/2017	10/23/2017	0.0313	27.76	88.703	0.50159744	11.2735758655288	501.59744
Col-0	Col_P1	9/25/2017	10/23/2017	0.0429	31.87	74.293	0.37910574	13.4602183247412	379.10574
Col-0	Col_P1	9/20/2017	10/18/2017	0.0505	35.66	70.615	0.38046805	14.1612971748212	380.46805
Col-0	Col_P1	9/18/2017	10/16/2017	0.0372	27.76	74.629	0.44134897	13.3996167709603	441.34897
Col-0	Col_P1	9/18/2017	10/16/2017	0.035	24.88	71.095	0.46311688	14.0656867571559	463.11688
Col-0	Col_P1	9/22/2017	10/20/2017	0.0442	33.59	76.001	0.35828877	13.1577216089262	358.28877
Col-0	Col_P1	9/20/2017	10/18/2017	0.0332	24.63	74.197	0.4745345	13.4776338665984	474.5345
Col-0	Col_P2	9/18/2017	10/16/2017	0.0311	22.51	72.375	0.47588424	13.8169257340242	475.88424
Col-0	Col_P2	9/22/2017	10/20/2017	0.0401	29.05	72.438	0.39106779	13.8049090256495	391.06779
Col-0	Col_P2	9/22/2017	10/20/2017	0.0366	26.62	72.733	0.40784898	13.7489172727648	407.84898
Col-0	Col_P2	9/25/2017	10/23/2017	0.0395	28.1	71.135	0.39125432	14.0577774653827	391.25432
Col-0	Col_P2	9/25/2017	10/23/2017	0.0549	41.14	74.94	0.32670972	13.3440085401655	326.70972
Col-0	Col_P2	9/20/2017	10/18/2017	0.0531	38.14	71.827	0.38614963	13.922341180893	386.14963
Col-0	Col_P2	9/18/2017	10/16/2017	0.0373	26.52	71.095	0.43504753	14.0656867571559	435.04753
Col-0	Col_P2	9/20/2017	10/18/2017	0.048	33.65	70.109	0.35755556	14.2635039723859	357.55556
Col-0	Col_P3	9/22/2017	10/20/2017	0.0332	24.76	74.571	0.46823658	13.410038755012	468.23658
Col-0	Col_P3	9/18/2017	10/16/2017	0.0241	19.19	79.63	0.53112033	12.5580811252041	531.12033
Col-0	Col_P3	9/25/2017	10/23/2017	0.0486	34.97	71.953	0.38645716	13.8979611690965	386.45716
Col-0	Col_P3	9/22/2017	10/20/2017	0.0413	31.74	76.847	0.43363416	13.0128697281612	433.63416
Col-0	Col_P3	9/20/2017	10/18/2017	0.0417	29.45	70.616	0.42991062	14.1610966353234	429.91062
Col-0	Col_P3	9/25/2017	10/23/2017	0.0542	39.16	72.253	0.33227105	13.8402557679266	332.27105
Col-0	Col_P3	9/18/2017	10/16/2017	0.0354	26.47	74.78	0.43990755	13.3725595078898	439.90755
Col-0	Col_P3	9/20/2017	10/18/2017	0.0455	32.04	70.41	0.43526474	14.2025280499929	435.26474
Col-0	Col_P4	9/20/2017	10/18/2017	0.0104	8.94	85.965	0.56354895	11.6326411911825	563.54895
Col-0	Col_P4	9/25/2017	10/23/2017	0.047	34.04	72.422	0.35744681	13.8079589075143	357.44681
Col-0	Col_P4	9/18/2017	10/16/2017	0.0287	21.76	75.816	0.46309788	13.1898280046428	463.09788
Col-0	Col_P4	9/20/2017	10/18/2017	0.0386	26.39	68.377	0.44441828	14.62480073709	444.41828
Col-0	Col_P4	9/18/2017	10/16/2017	0.0386	27.83	72.09	0.4418276	13.8715494520738	441.8276
Col-0	Col_P4	9/22/2017	10/20/2017	0.0366	30.28	82.725	0.45131644	12.0882441825325	451.31644
Col-0	Col_P4	9/25/2017	10/23/2017	0.0406	31.11	76.636	0.39565607	13.0486977399656	395.65607
Col-0	Col_P4	9/22/2017	10/20/2017	0.0358	27.27	76.173	0.45987811	13.1280112375776	459.87811
flm-3	flm-3_P1	9/25/2017	10/23/2017	0.0279	22.03	78.974	0.52329749	12.6623952186796	523.29749
flm-3	flm-3_P1	9/18/2017	10/16/2017	0.0245	18	73.453	0.5038961	13.6141478224171	503.8961
flm-3	flm-3_P1	9/22/2017	10/20/2017	0.0288	25.05	86.969	0.50031566	11.4983499867769	500.31566
flm-3	flm-3_P1	9/20/2017	10/18/2017	0.0375	27.36	72.95	0.45672727	13.7080191912269	456.72727
flm-3	flm-3_P1	9/25/2017	10/23/2017	0.0427	34.02	79.667	0.390462	12.5522487353609	390.462
flm-3	flm-3_P1	9/18/2017	10/16/2017	0.0403	28.03	69.559	0.41755019	14.3762848804612	417.55019
flm-3	flm-3_P1	9/20/2017	10/18/2017	0.0388	32.16	82.883	0.42666354	12.0652003426517	426.66354
flm-3	flm-3_P1	9/22/2017	10/20/2017	0.0563	40.61	72.14	0.34345228	13.8619351261436	343.45228
flm-3	flm-3_P2	9/22/2017	10/20/2017	0.0326	24.45	75.002	0.47016174	13.332977787259	470.16174

flm-3	flm-3_P2	9/20/2017	10/18/2017	0.0297	27	90.896	0.59779614	11.0015842281289	597.79614
flm-3	flm-3_P2	9/25/2017	10/23/2017	0.0297	23.61	79.492	0.45699418	12.5798822523021	456.99418
flm-3	flm-3_P2	9/22/2017	10/20/2017	0.0489	36.79	75.225	0.3508087	13.2934529744101	350.8087
flm-3	flm-3_P2	9/20/2017	10/18/2017	0.0374	31.27	83.607	0.4902771	11.9607209922614	490.2771
flm-3	flm-3_P2	9/18/2017	10/16/2017	0.0375	29.44	78.497	0.368	12.7393403569563	368
flm-3	flm-3_P2	9/18/2017	10/16/2017	0.0366	30.76	84.042	0.45628415	11.8988124985126	456.28415
flm-3	flm-3_P2	9/25/2017	10/23/2017	0.0342	30.25	88.451	0.42131845	11.3056946784095	421.31845
flm-3	flm-3_P3	9/18/2017	10/16/2017	0.0228	16.96	74.381	0.52472089	13.4442935695944	524.72089
flm-3	flm-3_P3	9/20/2017	10/18/2017	0.0389	29.76	76.516	0.43935499	13.0691620053322	439.35499
flm-3	flm-3_P3	9/25/2017	10/23/2017	0.039	28.25	72.43	0.41818182	13.8064337981499	418.18182
flm-3	flm-3_P3	9/25/2017	10/23/2017	0.0486	34.53	71.056	0.33127572	14.07340689034	331.27572
flm-3	flm-3_P3	9/18/2017	10/16/2017	0.0325	24.86	76.495	0.46461538	13.0727498529316	464.61538
flm-3	flm-3_P3	9/22/2017	10/20/2017	0.0318	25.31	79.607	0.48684963	12.5617093974148	486.84963
flm-3	flm-3_P3	9/20/2017	10/18/2017	0.0467	34.28	73.401	0.42164688	13.6237925913816	421.64688
flm-3	flm-3_P3	9/22/2017	10/20/2017	0.0361	29.49	81.703	0.43037018	12.2394526516774	430.37018
flm-3	flm-3_P4	9/25/2017	10/23/2017	0.0303	23.67	78.127	0.4950495	12.7996723283884	495.0495
flm-3	flm-3_P4	9/18/2017	10/16/2017	0.0383	26.51	69.204	0.43816758	14.4500317900699	438.16758
flm-3	flm-3_P4	9/22/2017	10/20/2017	0.0309	25.62	82.922	0.48543689	12.0595258194448	485.43689
flm-3	flm-3_P4	9/25/2017	10/23/2017	0.0397	27.04	68.108	0.40141974	14.6825629881952	401.41974
flm-3	flm-3_P4	9/22/2017	10/20/2017	0.0372	28.72	77.192	0.36119257	12.954710332677	361.19257
flm-3	flm-3_P4	9/18/2017	10/16/2017	0.032	22.92	71.634	0.484375	13.9598514671804	484.375
flm-3	flm-3_P4	9/20/2017	10/18/2017	0.0424	30.72	72.452	0.43578473	13.802241484017	435.78473
flm-3	flm-3_P4	9/20/2017	10/18/2017	0.0415	30.56	73.647	0.41861993	13.5782856056594	418.61993
rec099_Can	rec099_10	9/22/2017	10/20/2017	0.034	28.55	83.975	0.49064171	11.9083060434653	490.64171
rec099_Can	rec099_10	9/25/2017	10/23/2017	0.0466	40.63	87.189	0.35446742	11.469336728257	354.46742
rec099_Can	rec099_10	9/20/2017	10/18/2017	0.0396	33.26	83.992	0.48243802	11.9058957996	482.43802
rec099_Can	rec099_10	9/22/2017	10/20/2017	0.03	26.64	88.787	0.53566667	11.2629101107144	535.66667
rec099_Can	rec099_10	9/25/2017	10/23/2017	0.0419	31.83	75.964	0.40160555	13.1641303775473	401.60555
rec099_Can	rec099_10	9/20/2017	10/18/2017	0.0357	29.71	83.233	0.52559206	12.0144654163613	525.59206
rec099_Can	rec099_10	9/18/2017	10/16/2017	0.0396	30.96	78.179	0.44605142	12.7911587510713	446.05142
rec099_Can	rec099_10	9/18/2017	10/16/2017	0.0428	32.35	75.594	0.45433305	13.2285631134746	454.33305
rec099_Can	rec099_11	9/22/2017	10/20/2017	0.0313	27.61	88.218	0.5634621	11.3355551021334	563.4621
rec099_Can	rec099_11	9/25/2017	10/23/2017	0.0308	31.75	103.084	0.50708383	9.70082651041869	507.08383
rec099_Can	rec099_11	9/20/2017	10/18/2017	0.0411	32.57	79.237	0.45963282	12.6203667478577	459.63282
rec099_Can	rec099_11	9/25/2017	10/23/2017	0.0353	31.78	90.035	0.47437548	11.1067918031876	474.37548
rec099_Can	rec099_11	9/22/2017	10/20/2017	0.0406	33.37	82.195	0.3929691	12.1661901575522	392.9691
rec099_Can	rec099_11	9/20/2017	10/18/2017	0.0492	37.67	76.566	0.43717664	13.0606274325419	437.17664
rec099_Can	rec099_11	9/18/2017	10/16/2017	0.0412	32.52	78.923	0.46910856	12.6705776516351	469.10856
rec099_Can	rec099_11	9/18/2017	10/16/2017	0.0522	38.97	74.646	0.38714734	13.3965651207031	387.14734
rec099_Can	rec099_12	9/20/2017	10/18/2017	0.0364	31.81	87.385	0.53296703	11.4436116038222	532.96703
rec099_Can	rec099_12	9/22/2017	10/20/2017	0.0315	26.23	83.28	0.50995671	12.0076849183477	509.95671
rec099_Can	rec099_12	9/18/2017	10/16/2017	0.0301	25.07	83.274	0.52129266	12.0085500876624	521.29266
rec099_Can	rec099_12	9/25/2017	10/23/2017	0.0477	36.57	76.674	0.35201067	13.0422307431463	352.01067
rec099_Can	rec099_12	9/25/2017	10/23/2017	0.046	37.69	81.94	0.38873518	12.2040517451794	388.73518
rec099_Can	rec099_12	9/22/2017	10/20/2017	0.0394	32.57	82.663	0.41716659	12.0973107678163	417.16659
rec099_Can	rec099_12	9/18/2017	10/16/2017	0.0298	23.61	79.244	0.4966443	12.6192519307455	496.6443

rec099_Can	rec099_12	9/20/2017	10/18/2017	0.0368	28.88	78.486	0.50842391	12.7411258058762	508.42391
rec099_Col	rec099_13	9/20/2017	10/18/2017	0.0483	36.25	75.06	0.42499529	13.3226751931788	424.99529
rec099_Col	rec099_13	9/18/2017	10/16/2017	0.0241	19.84	82.321	0.49377593	12.1475686641319	493.77593
rec099_Col	rec099_13	9/18/2017	10/16/2017	0.0437	34.24	78.351	0.43166216	12.7630789651696	431.66216
rec099_Col	rec099_13	9/25/2017	10/23/2017	0.0389	31.53	81.063	0.42603412	12.3360842801278	426.03412
rec099_Col	rec099_13	9/20/2017	10/18/2017	0.0333	26.77	80.393	0.50778051	12.4388939335514	507.78051
rec099_Col	rec099_13	9/22/2017	10/20/2017	0.0362	29.34	81.049	0.51657459	12.3382151537959	516.57459
rec099_Col	rec099_13	9/25/2017	10/23/2017	0.0493	35.99	72.995	0.38244514	13.6995684635934	382.44514
rec099_Col	rec099_13	9/22/2017	10/20/2017	0.0494	38.62	78.174	0.35112256	12.7919768721058	351.12256
rec099_Col	rec099_14	9/22/2017	10/20/2017	0.033	26.09	79.074	0.52699725	12.646381870147	526.99725
rec099_Col	rec099_14	9/25/2017	10/23/2017	0.0332	30.55	92.031	0.55750274	10.8659038802143	557.50274
rec099_Col	rec099_14	9/25/2017	10/23/2017	0.0441	42.01	95.252	0.33992991	10.4984672237853	339.92991
rec099_Col	rec099_14	9/22/2017	10/20/2017	0.0387	31.32	80.926	0.47968053	12.3569680943084	479.68053
rec099_Col	rec099_14	9/18/2017	10/16/2017	0.0496	38.62	77.86	0.416239	12.8435653737478	416.239
rec099_Col	rec099_14	9/20/2017	10/18/2017	0.0454	41.69	91.838	0.44603524	10.8887388662645	446.03524
rec099_Col	rec099_14	9/20/2017	10/18/2017	0.0367	29.11	79.327	0.48228883	12.6060483820137	482.28883
rec099_Col	rec099_14	9/18/2017	10/16/2017	0.0455	34.04	74.813	0.45234765	13.366660874447	452.34765
rec099_Col	rec099_15	9/20/2017	10/18/2017	0.0455	34.78	76.431	0.46053946	13.0836964059086	460.53946
rec099_Col	rec099_15	9/25/2017	10/23/2017	0.051	40.6	79.612	0.30089127	12.5609204642516	300.89127
rec099_Col	rec099_15	9/22/2017	10/20/2017	0.053	39.19	73.941	0.35608919	13.5242963984799	356.08919
rec099_Col	rec099_15	9/25/2017	10/23/2017	0.0453	31.79	70.181	0.38571142	14.2488707769909	385.71142
rec099_Col	rec099_15	9/18/2017	10/16/2017	0.0585	43.53	74.414	0.37622378	13.4383314967614	376.22378
rec099_Col	rec099_15	9/18/2017	10/16/2017	0.0446	30.96	69.42	0.42376682	14.4050705848459	423.76682
rec099_Col	rec099_15	9/22/2017	10/20/2017	0.0475	35.36	74.434	0.3554067	13.4347206921568	355.4067
rec099_Col	rec099_15	9/20/2017	10/18/2017	0.0458	38.48	84.009	0.45533942	11.903486531205	455.33942
rec099_Col	rec099_1A	9/25/2017	10/23/2017	0.0381	32.19	84.489	0.49797184	11.8358602895051	497.97184
rec099_Col	rec099_1A	9/22/2017	10/20/2017	0.0619	47.05	76.013	0.32706712	13.1556444292424	327.06712
rec099_Col	rec099_1A	9/18/2017	10/16/2017	0.0327	24.27	74.233	0.50430915	13.4710977597564	504.30915
rec099_Col	rec099_1A	9/18/2017	10/16/2017	0.0318	23.58	74.138	0.49056604	13.4883595457121	490.56604
rec099_Col	rec099_1A	9/20/2017	10/18/2017	0.0558	44.95	80.564	0.36102965	12.4124919318802	361.02965
rec099_Col	rec099_1A	9/25/2017	10/23/2017	0.0359	26.93	75.028	0.43530008	13.3283574132324	435.30008
rec099_Col	rec099_1A	9/20/2017	10/18/2017	0.0523	43.76	83.662	0.39196941	11.9528579283307	391.96941
rec099_Col	rec099_1A	9/22/2017	10/20/2017	0.0707	49.94	70.641	0.30705928	14.1560849931343	307.05928
rec099_Can	rec099_1B	9/25/2017	10/23/2017	0.0428	34.74	81.166	0.36406117	12.3204297365892	364.06117
rec099_Can	rec099_1B	9/18/2017	10/16/2017	0.0306	24.25	79.26	0.53178847	12.6167045167802	531.78847
rec099_Can	rec099_1B	9/18/2017	10/16/2017	0.0284	22.99	80.943	0.5003201	12.3543728302633	500.3201
rec099_Can	rec099_1B	9/22/2017	10/20/2017	0.0315	28.6	90.785	0.48917749	11.0150355234896	489.17749
rec099_Can	rec099_1B	9/22/2017	10/20/2017	0.0476	39.19	82.337	0.34816654	12.1452081081409	348.16654
rec099_Can	rec099_1B	9/25/2017	10/23/2017	0.0318	33.07	103.979	0.57175529	9.61732657555853	571.75529
rec099_Can	rec099_1B	9/20/2017	10/18/2017	0.0467	37.78	80.894	0.39497761	12.3618562563355	394.97761
rec099_Can	rec099_1B	9/20/2017	10/18/2017	0.0453	37.28	82.292	0.44210315	12.1518495114956	442.10315

date_t0growth: End of stratification i.e. date at which plants have been transferred to the greenhouse; date_PN1: Date at which photosynthesis has been measured; DM_Leaves: Dry mass of the leaf blades; Leaves_area_scan: Total leaf area from dissected and scanned rosettes; SLA: Specific leaf area at harvesting [SLA = Leaf area / leaf DM]; Photo_DM_Leaves: Net photosynthetic rate per unit leaf DM [PN raw / Leaf DM]; LMA: Leaf mass per area [1/SLA*1000]; Amass: Net photosynthetic rate per unit leaf DM [PN raw / Leaf DM*1000]

Supplementary Table 3. List of primers used in this study.

purpose	gene/element	primer name	sequence
gateway cloning	gFLM	AttB1-pFLM	GGGGACAAGTTTGTACAAAAAAGCAGGCTctcgaacaagtttttttcttctctctaatcga
gateway cloning	gFLM	AttB2-FLMStop	GGGGACCACTTTGTACAAGAAAGCTGGGTACCAAAACATGAAACAGAGTTCAAAAGCTG
PCR-mediated mutagenesis	gFLM	FLM_hyb-A-F	TCTGGATGCAGTTTTTGGTGTTA
PCR-mediated mutagenesis	gFLM	FLM_hyb-A-R	TAACACCAAAAACTGCATCCAGA
qPCR	PP2A	qPP2A-F	TTTGTGAAGCTGTAGGACCG
qPCR	PP2A	qPP2A-R	CGAGTTCAGGGTTTAAAATGCG
qPCR	FLM	qFLM-F3	TCCAAACGACGCAATGGTCTCATCGA
qPCR	FLM	qFLM-R3b	TTCATAACGATCAATGATCTTGGAAATG
PCR	FLM	qFLM-R3d	TCCGGCTTGAACAGCGCTTCTATC
pTOPO isoforms cloning	FLM	FLM-cDNA-Fw	ATGGGAAGAAGAAAAATCGAGAT
pTOPO isoforms cloning	FLM	FLM-cDNA-Rev	CTAATTGAGCAGCGGGAGAGTCT
