# Supplementary material for: Natural variation at FLM splicing has pleiotropic effects modulating ecological strategies in Arabidopsis thaliana
Source: Nat Commun. 2020 Aug 18;11:4140. doi: 10.1038/s41467-020-17896-w (PMC7435183; doi:10.1038/s41467-020-17896-w)
Supplement: Supplementary file 3 — Reporting Summary [file 41467_2020_17896_MOESM3_ESM.pdf]

nature research

Corresponding author(s): Hanemian & Loudet

Last updated by author(s): Jun 29, 2020

Reporting Summary

Nature Research wishes to improve the reproducibility of the work that we publish. This form provides structure for consistency and transparency in reporting. For further information on Nature Research policies, see our Editorial Policies and the Editorial Policy Checklist.

Statistics

For all statistical analyses, confirm that the following items are present in the figure legend, table legend, main text, or Methods section.

n/a	Confirmed	
☐	☒	The exact sample size (n) for each experimental group/condition, given as a discrete number and unit of measurement
☒	☐	A statement on whether measurements were taken from distinct samples or whether the same sample was measured repeatedly
☐	☒	The statistical test(s) used AND whether they are one- or two-sided<br>*Only common tests should be described solely by name; describe more complex techniques in the Methods section.*
☐	☒	A description of all covariates tested
☒	☐	A description of any assumptions or corrections, such as tests of normality and adjustment for multiple comparisons
☐	☒	A full description of the statistical parameters including central tendency (e.g. means) or other basic estimates (e.g. regression coefficient) AND variation (e.g. standard deviation) or associated estimates of uncertainty (e.g. confidence intervals)
☐	☒	For null hypothesis testing, the test statistic (e.g. F, t, r) with confidence intervals, effect sizes, degrees of freedom and P value noted<br>*Give P values as exact values whenever suitable.*
☒	☐	For Bayesian analysis, information on the choice of priors and Markov chain Monte Carlo settings
☒	☐	For hierarchical and complex designs, identification of the appropriate level for tests and full reporting of outcomes
☒	☐	Estimates of effect sizes (e.g. Cohen's d, Pearson's r), indicating how they were calculated

Our web collection on statistics for biologists contains articles on many of the points above.

Software and code

Policy information about availability of computer code

Data collection	No software used
Data analysis	R 3.2.3

For manuscripts utilizing custom algorithms or software that are central to the research but not yet described in published literature, software must be made available to editors and reviewers. We strongly encourage code deposition in a community repository (e.g. GitHub). See the Nature Research guidelines for submitting code & software for further information.

Data

Policy information about availability of data

All manuscripts must include a data availability statement. This statement should provide the following information, where applicable:

- Accession codes, unique identifiers, or web links for publicly available datasets
- A list of figures that have associated raw data
- A description of any restrictions on data availability

Data supporting the findings of this work are available within the paper and its Supplementary Information files. The source data underlying Figures 1a-d, 2a, 3, 5a, 6, and Supplementary Figure 1b, 3a-b, 5, and 6, are provided as a Source Data file. The data used for Figure 4a are supplied as Supplementary Table 2. The data used for Figure 5b-c are supplied as Supplementary Data 3. The data used for Supplementary Figure 1a are supplied as Supplementary Data 5. The data used for the figure 4b can be found in the GLOPNET database (https://www.try-db.org/TryWeb/Home.php) for the 'All species' and 'Herbaceous' datasets, and in INRAE data repository (https://doi.org/10.15454/B3W0OS) for the 'A. thaliana accessions' dataset.
R scripts customized for analyzing data are available from the authors upon reasonable request.

Field-specific reporting

Please select the one below that is the best fit for your research. If you are not sure, read the appropriate sections before making your selection.

☒ Life sciences ☐ Behavioural & social sciences ☐ Ecological, evolutionary & environmental sciences

For a reference copy of the document with all sections, see nature.com/documents/nr-reporting-summary-flat.pdf

Life sciences study design

All studies must disclose on these points even when the disclosure is negative.

Sample size	Sample size was determined empirically to ensure statistical confidence in the results obtained, depending on the expected effect size and our knowledge of the heritability of the trait based essentially on previous experience (Tisné et al., The Plant Journal, 2013; Marchadier et al., PLoS Genetics, 2019)
Data exclusions	no data points were excluded
Replication	All the experiments were replicated at least 2 times to verify the reproducibility with all attempts successful, except the experiments presented in Fig 3 & 4 and Supplementary Fig 4 due to the difficulty and the length of such experiment. This was compensated by a large number of samples
Randomization	Samples were randomized in trays or in the phenotyping robot before measurements.
Blinding	Most of the experiments were done blindingly as the data were collected in a phenotyping platform or numbers were attributed to plant genotypes.

Reporting for specific materials, systems and methods

We require information from authors about some types of materials, experimental systems and methods used in many studies. Here, indicate whether each material, system or method listed is relevant to your study. If you are not sure if a list item applies to your research, read the appropriate section before selecting a response.

Materials & experimental systems

n/a	Involved in the study
☒	☐ Antibodies
☒	☐ Eukaryotic cell lines
☒	☐ Palaeontology and archaeology
☒	☐ Animals and other organisms
☒	☐ Human research participants
☒	☐ Clinical data
☒	☐ Dual use research of concern

Methods

n/a	Involved in the study
☒	☐ ChIP-seq
☒	☐ Flow cytometry
☒	☐ MRI-based neuroimaging
