# Supplementary material for: Natural variation at FLM splicing has pleiotropic effects modulating ecological strategies in Arabidopsis thaliana
Source: Nat Commun. 2020 Aug 18;11:4140. doi: 10.1038/s41467-020-17896-w (PMC7435183; doi:10.1038/s41467-020-17896-w)
Supplement: Supplementary file 4 — Description of Additional Supplementary Files [file 41467_2020_17896_MOESM4_ESM.pdf]

Description of Additional Supplementary Files

File name: Supplementary Data 1
Description: Genomic sequence of FLM in Col-0 and Can-0

File name: Supplementary Data 2
Description: All the accessions carrying SNP28958 were extracted using polymorph (http://tools.1001genomes.org/polymorph/)

File name: Supplementary Data 3
Description: Geographic and phenotypic data of 186 accessions from Iberian Peninsula.
These data were obtained from Mendez-Vigo et al 2011 and Vidigal et al 2016.
From the Supplemental Table S8 of Mendez-Vigo et al 2011: Phenotypic values of all traits and accessions. OVR: Obligate vernalization requirement; LN: leaf number without vernalization ; FT: flowering time without vernalization; LN/FT: rate of leaf production without vernalization; VNF: Percentage of non-flowering plants after vernalization; VLN: leaf number with vernalization; VFT: flowering time with vernalization; VLN/VFT: rate of leaf production with vernalization; SLN: vernalization sensitivity for leaf number; SFT: vernalization sensitivity for flowering time; ND: Non-determined.
From the Supporting Information Table S1 of Vidigal et al 2016: Geographic and phenotypic information of Iberian A. thaliana accessions. Long: Longitude; Lat: Latitude; Alt (m.a.s.l.): Altitude (meter above sea level); DSDS10, days of seeds dry storage required to reach 10% of germination; DSDS50, days of seeds dry storage required to reach 50% of germination; DSDS90, days of seeds dry storage required to reach 90% of germination; GAS: germination after stratification; t10, time required for 10% of viable seeds to germinate; t50, time required for 50% of viable seeds to germinate; U8416, uniformity of germination is the time interval between 84% and 16% of viable seeds to germinate; SS: seed size; FT: flowering time. DOG1-PP1 adn DOG1-PP2: DOG1 expression analysed with PP1 and PP2 pairs of primers and normaliced on At4g12590 and At4g34270.
ND: Non-determined. 1: Number of seed replicates used for estimation of seed traits.

File name: Supplementary Data 4
Description: Genotype of the recombinant used for the fine-mapping
Markers starting with "c1" are SNP markers used for the QTL mapping
A means Col-0. B means Can-0. H means heterozygote.
If a genotype is framed by a squared bracket, it means that it was deduced from a genotyping done in a previous generation

File name: Supplementary Data 5
Description: Phenotypic values used to perform the QTL mapping
RIL ID: the numbers correspond to the RIL lines used (more information here http://publiclines.versailles.inra.fr/)
Cond: watering condition, here WW (Well Watered) corresponds to 60% of soil water content
The other columns correspond to the phenotypes measured in 2 independent experiments performed on the Phenoscope
