# Supplementary material for: Natural variation at FLM splicing has pleiotropic effects modulating ecological strategies in Arabidopsis thaliana
Source: Nat Commun. 2020 Aug 18;11:4140. doi: 10.1038/s41467-020-17896-w (PMC7435183; doi:10.1038/s41467-020-17896-w)
Supplement: Supplementary file 2 — Peer Review [file 41467_2020_17896_MOESM2_ESM.pdf]

Reviewers' comments:

Reviewer #1 (Remarks to the Author):

In this manuscript, entitled Splicing natural variation at FLM induces 1 synergistic pleiotropy in Arabidopsis thaliana‟, Hanemian and co-authors report the dissection of a QTL controlling the rosette growth difference shown by Can-0 and Col-0, two A. thaliana genotypes. The fine-mapping of a QTL in chromosome 1, followed by a thorough functional validation (complementation by allelic test and by transformation) reveals that this variation is encoded in the FLM locus. Despite the absence of coding variation, directed mutagenesis demonstrates that a variant affecting splicing causes the modification. That splicing affects function at the FLM locus had already been demonstrated, but the authors work further to show that this natural variant not only affects rosette growth and flowering time, it also alters the components of the leaf economic spectrum. A modification of the function of FLM appears to be sufficient to shift the leaf economic spectrum by a few percent towards increased resource-acquisitive leaf physiology. To my knowledge, this is the first time that a flowering time allele is explicitly related to a phenotypic measure of central ecological relevance. The authors further show that the loss of function allele of Can-0 is shared by a number of other Iberian genotypes, and the distribution of the alleles associates with areas where temperature is relatively less homogeneous. The results presented in this manuscript are excellent, and the association between flowering and LES very novel and convincing.The elaboration on the putative adaptive relevance of the variant is an interesting addition that adds relevance to the work. I therefore strongly recommend this manuscript for publication. I think at this point that the discussion needs some work.

Here are several points that the final version should improve.
1) I am confused about how the authors interpret temperature effects. The QTL at FLM seems to be dependent on mean temperature and the polymorphism seems to associate with temperature heterogeneity. Why should that imply that its action is dependent on the amplitude of temperature fluctuations? Differences between 16 and 23 degrees are well within the realm of the daily temperature fluctuations one expects, no? Would it not be straightforward to validate this hypothesis?
2) Why are Col-0 and flm-3 so far off the spectrum measured within A. thaliana and across species (Fig.4b)? I think there is something unrealistic there. Why should thaliana display the LES range of all herbaceous species (it is not a succulent)?
3) Is it necessary to arm-wave around the role of FLM on WUE? I believe it is more relevant to discuss the link with LES. What does it mean? Why can one believe that this have any ecological relevance? Is LES related to fitness and how?
4) Origin of the Can allele. Which of the two alleles is ancestral? Why not use the other Brassicaceae species to determine the ancestral state?
5) Relict origin: while I understand that the authors are referring to this classification to describe the history of the Can allele, the literature on relics is unclear as to what really defines relicts. A more generic conclusion is that the Can-0 and the Col-0 allele have distinct admixed origins. In this context, it is not straightforward to determine whether the Can-0 allele is adaptive (the association with temperature fluctuations suggests so, but this would have to be experimentally confirmed).
6) The last paragraph must be rewritten. I think the authors are somewhat confused about several concepts. Studies show that mutations with an intermediate pleiotropic effect are more likely to be consistently selected when seasonal conditions fluctuate. The authors show some pleiotropic effects of FLM, but the pattern of selection of each of these traits is unclear. Is flowering time variation independent of the LES, or is the LES a trait integrating variation across molecular processes, at the organ level? What I mean here is that the authors would not describe the effect of the mutation on splicing and flowering time as being pleiotropic. They are causal. As long as the causality on LES is not determined, it is a stretch to highlight the case studies by the authors as an example of adaptive (ie synergistic) pleiotropic variant. Can the two traits evolve independently as well (in which case one could study whether the pleiotropic mutation is more adaptive than a none pleiotropic mutation affecting only FT and not LES or only LES and not FT)? I understand that such follow-up work is not in the realm of the present manuscript, but I think the manuscript would be improved if it were

refocused on the significance of pleiotropy, making clear that whether it is causality that links these traits, or a synergistic association between two traits whose genetic basis can otherwise be independent is not known.
7) As a consequence, I don't think the authors bring evidence of synergistic pleiotropy. So the author should either make their point more clearly or change their title.

Miscellaneous comments:
Line 169: what is "higher" growth? Faster growth? Higher final PRA?
Line 252-253: I don't think the word "trigger" is appropriate. May be something like "The effect of FLM loss of function on LMA reaches 2-3% of the total variance….". In addition, since LMA and Amass being way out of the range normally reported in plants, it is unclear whether the effect reported here is realistic.
Line 283: I appreciate that the authors are being cautious on this conclusion. Their p-value is not massively significant, and such correlation can also arise from outliers of population structure. This hypothesis should be validated with ad-hoc experiment manipulating temperature fluctuations.
Line 355-356: not clear why mentioning neo-functionalization adds to the discussion. I think this paragraph is not very useful.
Line 363: P5CS1 splicing: please check this work carefully, the alternative splicing affects a transcript that is not necessary for function.
Line 401: this sentence is confusing. Please rewrite.
Line 413: reciprocal planting experiments? Where? Statement is unclear. I think the authors mean a common garden across environments differing in temperature fluctuations and precipitations.
Fig. 4b: why so many points for Col-0/flm3? Are the 30 replicates shown here? Would a mean with error bars be more appropriate? The legend needs to be clarified.

Reviewer #2 (Remarks to the Author):

The middle of this paper (Results) describes a nice genetic dissection of some growth traits differences between 2 Arabidopsis lines. Hanemian et al. first grew RILs from the Can-0 and Col-0 cross and performed a QTL analysis. They then watched the major QTL segregate in the progeny of a residually heterozygous RIL, confirming the phenotypic effect. The QTL contained FLOWERING LOCUS M (FLM), so the authors went to nulls for this gene. Next, they performed a quantitative complementation assay in F1 hybrid plants to mutant flm-3. Finally, Hanemian et al. transformed the flm-3 mutant with the indicated sequence from both parental lines. In aggregate, these data show that allelic variation at FLM is responsible for growth and color differences between Can-0 and Col-0 owing to the QTL on chromosome 1. Subsequent work suggests that a SNP creates a splice site difference, causing the phenotype. In general, the work is a compelling demonstration of the power of forward genetics in Arabidopsis.

The introduction needs a complete re-write. Despite much lingo and many broad statements, I could not tell what the paper was actually about until I read the results section (which is very clear). Regarding the motivation for the study, the fact that a mutation to a MADS-box transcription factor has effects on multiple phenotypes is not remotely surprising. Any mutation that affects growth or allocation will affect numerous plant dimensions through time. However, the fact that the Quantitative Trait Nucleotide (QTN) is a splice variant is notable discovery. While not unprecedented (line 359 onward), the result bears directly on the general question of what sorts of mutations make a QTN. This would be a better focus for the Introduction/Discussion.

Reviewer #3 (Remarks to the Author):

The manuscript by Haneman et al describes how natural variation at the FLOWERING LOCUS M (FLM) gene contributes to pleiotropy in Arabidopsis. The authors mapped QTLs for several growth related traits, which turned out to be mapping to the FLM locus. When they analysed the FLM locus in strains that differed in the alleles, the underlying molecular mechanism hinted at changes in splicing. The authors go to length to try and establish that variation in splicing contributes to this variation across multiple growth related traits. The authors claim that Natural variation in FLM splicing mediates synergistic pleiotropy in Arabidopsis. The authors show that the alleles of interest have an interpretable geographic distribution indicative of adaptive significance. Overall the story is interesting, as it goes beyond flowering and it would be of interest to plant biologists and evolutionary biologists. However the study suffers from few issues, which I have listed below, addressing which would substantially improve this manuscript.

This manuscript suffers severely from a chronological effect of experiments. In 2013/14, there was Nature paper published by Pose et al, which proposed a model based on an assumption that the primary transcripts arising from FLM are FLM-beta and FLM-delta. This assumption was incorrect and this mis-led the field and several researchers on a wrong track. This paper also appears to be one of those mis-led by this story and that is reflected in their interpretation. When I look at the data they present it is very clear, what is really happening. In Col-0 you have primarily FLM-beta. In Can-0, you have a new splice acceptor site that is strong enough to outcompete the normal splice site that would have resulted in FLM-beta. Therefore, with the new splicing instead of AG1 (i.e., splice acceptor that is in front of Exon2) the new splice site is used resulting in a novel transcript, that contains a premature stop codon. The authors are too influenced by the beta/delta model presumably due to historical reasons, and fail to see this. As far as I could see their data clearly shows that the primary effect is loss of the normal (FLM-beta) transcript and formation of a novel transcript with a premature stop codon due to a hot splice acceptor site. There is no need at all to discuss their work in the context of FLM-delta in this paper. This is the primary problem with this paper and it requires re-writing the paper with the current knowledge. The authors presumably are tip-toeing around this issue, which is not very helpful.

I have given some clear suggestions below.

Fig 1A:

rHIF068: The legend says it is fixed for FLM. The genotype panel indicates that orange is Col and Blue is Can. Then what do the colours indicate in rHIF68? Does this mean that rHIF068 that is fixed for Col and Can alleles for FLM do not differ in their phenotypes? Is this correct? Or rHIF68 is fixed for Col allele? This makes not much sense.

Fig 1B & D.
16ºC panel figure.. Did all 21 plants flowered on day 30 in the flm mutant background? Is this what the figure shows? How was days to flowering measured. This lack of variation is a bit strange… I really have difficulties to believe that all 15 plants in Col flowered on day 54 and all 30 plants of flm flowered on day 47 or something. Please explain.

Fig 1C: What is this significance for? The data discussed in lines 181 and 183 is somewhat inconsistent with the figure. Please double check this analysis and its interpretation.

Lines: 194 to 195. Rephrase this.. This idea of discussing FLM splicing in the context of FLM beta and delta leads to fundamental confusion in the manuscript. It is better to state that FLM

splicing/expression

Fig 2 and Fig S3. It think both these figures can be removed. They do not really add any value. It is pretty clear from this work as well as from earlier works that such analysis would lead to misleading conclusions. The authors have done a lot of work. But this does not help either the paper or the story itself. I suggest the authors to directly discuss the sequencing of full-length cDNA clones and remove this from the manuscript. The entire section of lines 194 to 213 can be removed. I believe all these discussions with exon2 vs exon 3 etc are not very useful. The authors can simply state that temperature affects FLM splicing and then present their cloning data. I suggest to make Fig S4 as the main fig S2, which can easily drive the point.

Lines 231 and 232: We have no idea whether the promotion of growth has got anything to do with these alleles. Should be rephrased to say that there was no differences in PRA were observed between plants harbouring either allele.

Lines 249-250: Unclear … what is being shown here and what does that mean?

Lines: 241-254: I have no idea what is meant by this. E.g., “variation observed across all plant species”???

Lines: 285-300: Unclear. I am not sure whether this data is convincing enough.

Lines 327-329: “which is expressed at a substantial level in many Arabidpsis strains”? where is this data from… I do not believe any paper has shown this clearly. I suggest to remove this.

Lines 338: Remove in exon 3 etc.. A premature stop codon is a premature stop codon. End of story.

Lines 344-346 and the full line of argument: This is incorrect to state. Nd-1 and Ei-6 carries full deletion of the FLM locus. I am not sure how appropriate is the line of argument made in this paragraph.

Fig. S5B: Are there 2 Col-0 lanes?

Hanemian et al.
Nature Communications

Point by point response to reviewers' comments

>>> Please refer to the track changes version of the text submitted to see the modifications in the text

Reviewer #1 (Remarks to the Author):

In this manuscript, entitled Splicing natural variation at FLM induces 1 synergistic pleiotropy in Arabidopsis thaliana“, Hanemian and co-authors report the dissection of a QTL controlling the rosette growth difference shown by Can-0 and Col-0, two A. thaliana genotypes. The fine-mapping of a QTL in chromosome 1, followed by a thorough functional validation (complementation by allelic test and by transformation) reveals that this variation is encoded in the FLM locus. Despite the absence of coding variation, directed mutagenesis demonstrates that a variant affecting splicing causes the modification. That splicing affects function at the FLM locus had already been demonstrated, but the authors work further to show that this natural variant not only affects rosette growth and flowering time, it also alters the components of the leaf economic spectrum. A modification of the function of FLM appears to be sufficient to shift the leaf economic spectrum by a few percent towards
increased resource-acquisitive leaf physiology. To my knowledge, this is the first time that a flowering time allele is explicitly related to a phenotypic measure of central ecological relevance. The authors further show that the loss of function allele of Can-0 is shared by a number of other Iberian genotypes, and the distribution of the alleles associates with areas where temperature is relatively less homogeneous.
The results presented in this manuscript are excellent, and the association between flowering and LES very novel and convincing.The elaboration on the putative adaptive relevance of the variant is an interesting addition that adds relevance to the work. I therefore strongly recommend this manuscript for publication. I think at this point that the discussion needs some work.

Here are several points that the final version should improve.
1) I am confused about how the authors interpret temperature effects. The QTL at FLM seems to be dependent on mean temperature and the polymorphism seems to associate with temperature heterogeneity. Why should that imply that its action is dependent on the amplitude of temperature fluctuations? Differences between 16 and 23 degrees are well within the realm of the daily temperature fluctuations one expects, no? Would it not be straightforward to validate this hypothesis?

>>> On the one hand, we used the known temperature-dependent impact of FLM on flowering time to validate our QTL (Fig1B) and subsequently on the other hand, we observed an association between the causal polymorphism in the FLM gene and temperature heterogeneity (Fig5C). We didn't mean to imply that FLM action is dependent on the temperature heterogeneity, but that allelic variation at FLM and its distribution may depend on this climatic variable. Still, we think the impact of daily temperature changes should not be confounded with temperature fluctuations across the plant cycle and development. Testing the impact and mode of action of temperature fluctuation consequences either on FLM splicing or flowering time would indeed require more careful experiments especially in growth chambers set at different temperature regimes, but this doesn't seem so straightforward to us as there are multitude of temperature fluctuations that could be tested.
We are not totally confident in our understanding of this specific question, so if we have missed the point of the reviewer here, please get back to us.

2) Why are Col-0 and flm-3 so far off the spectrum measured within A. thaliana and across species (Fig.4b)? I think there is something unrealistic there. Why should thaliana display the LES range of all herbaceous species (it is not a succulent)?

>>> The fact that our absolute values for these traits fell outside of the range previously observed across species and among A. thaliana accessions can be explained by the growth conditions in which plants from the 'FLM' dataset were measured (Fig. 4B). Indeed, plants were grown in the greenhouse under relatively low light intensity (ca. 65 µmol m^{-2} s^{-1} PPFD), compared to the natural accessions grown in the PHENOPSIS phenotyping platform under moderate light intensity for instance (ca. 195 µmol m^{-2} s^{-1} PPFD; Granier et al., 2006), or the interspecific data collected in natura under much higher (and fluctuating) light intensity. Still, the main point is that the relationship between these traits was conserved across these datasets ($p>0.05$).

3) Is it necessary to arm-wave around the role of FLM on WUE? I believe it is more relevant to discuss the link with LES. What does it mean? Why can one believe that this have any ecological relevance? Is LES related to fitness and how?

>>> To help focusing the discussion on LES as suggested by this reviewer, we have removed the discussion on WUE (L395-406 track-changes doc). We also agree that we had not explained with sufficient details the interest in detecting variation in the LES for plant adaptation. This has now been discussed and clarified in the 2nd paragraph of the discussion (resubmitted version).

4) Origin of the Can allele. Which of the two alleles is ancestral? Why not use the other Brassicaceae species to determine the ancestral state?

>>> This is a very good suggestion and we had already investigated this idea. Unfortunately, the lack of a clear haplotype underlying FLM-Can allele restricts the investigation to a single SNP position. We also aligned the sequences of the intron2 of FLM from A. lyrata, A. halleri, C. grandiflora, C. rubella, FLM-Can and FLM-Col. The A. thaliana sequences of this intron were much shorter than any of the other sequences making the comparison difficult. We also drew phylogenetic trees using genomic, coding and intron2 FLM sequences of the same species, but the A. thaliana always clustered altogether. Consequently we decided to describe this as an allele of ancient origin and not the ancestral allele (now L273), as we were unable to characterise it.

5) Relict origin: while I understand that the authors are referring to this classification to describe the history of the Can allele, the literature on relics is unclear as to what really defines relicts. A more generic conclusion is that the Can-0 and the Col-0 allele have distinct admixed origins. In this context, it is not straightforward to determine whether the Can-0 allele is adaptive (the association with temperature fluctuations suggests so, but this would have to be experimentally confirmed).

>>> Thanks for your remark. We have introduced even more caution now as to our use of relicts in the results and discussion and refer to the "generic conclusion" suggested by this reviewer (now L273 & L391). As for the adaptive character of variation at FLM, we have reviewed our text and we think we have now been careful in using this term and indeed we suggest experiments to confirm this.

6) The last paragraph must be rewritten. I think the authors are somewhat confused about several concepts. Studies show that mutations with an intermediate pleiotropic effect are more likely to be consistently selected when seasonal conditions fluctuate. The authors show some pleiotropic effects of FLM, but the pattern of selection of each of these traits is unclear. Is flowering time variation independent of the LES, or is the LES a trait integrating variation across molecular processes, at the organ level? What I mean here is that the authors would not describe the effect of the mutation on splicing and flowering time as being pleiotropic. They are causal. As long as the causality on LES is not determined, it is a stretch to highlight the case studies by the authors as an example of adaptive (ie synergistic) pleiotropic variant. Can the two traits evolve independently as well (in which case one could study whether the pleiotropic mutation is more adaptive than a none pleiotropic mutation affecting only FT and not LES or only LES and not FT)? I understand that such follow-up work is not in the realm of the present manuscript, but I think the manuscript would be improved if it were refocused on the significance of pleiotropy, making clear that whether it is causality that links these traits, or a synergistic association between two traits whose genetic basis can otherwise be independent is not known.

>>> We have modified the text at several instances to keep it clear that we did not determine the causality relationships between traits related to LES and flowering time (now L322-323). We also added to the discussion that genes impacting flowering time

were previously associated with LES, although the relationship between these is not resolved (see 2nd and 3rd paragraphs of the new version).

7) As a consequence, I don't think the authors bring evidence of synergistic pleiotropy. So the author should either make their point more clearly or change their title.

>>> ***We have introduced much more caution in our reference to synergistic pleiotropy and have in particular removed synergistic from our title and abstract. Whenever we refer to this mechanism, we state that this is only one possible explanation. As per point #3) of this reviewer, we also discuss in more details the ecological relevance of LES, giving more context to its relationship with development and adaptive role.***

Miscellaneous comments:
Line 169: what is "higher" growth? Faster growth? Higher final PRA?

>>> ***This has been changed for faster growth (now L152).***

Line 252-253: I don't think the word "trigger" is appropriate. May be something like "The effect of FLM loss of function on LMA reaches 2-3% of the total variance….".

>>> ***This has been corrected accordingly (now L225-227)***

In addition, since LMA and Amass being way out of the range normally reported in plants, it is unclear whether the effect reported here is realistic.

>>> ***See answer to point #2)***

Line 283: I appreciate that the authors are being cautious on this conclusion. Their p-value is not massively significant, and such correlation can also arise from outliers of population structure. This hypothesis should be validated with ad-hoc experiment manipulating temperature fluctuations.

>>> ***In this paragraph, we used the same type of statistical analysis as in the previous paragraph (now L240-246) i.e. taking into account the population structure. Regarding the p value, we inverted the 2 last sentences of the paragraph as it could be confusing (now L252-256). Reviewer1 is right, an experiment would be required to validate this hypothesis (now stated in L336-341).***

Line 355-356: not clear why mentioning neo-functionalization adds to the discussion. I think this paragraph is not very useful.

>>> ***The sentence mentioning the neofunctionalization has been removed.***

Line 363: P5CS1 splicing: please check this work carefully, the alternative splicing affects a transcript that is not necessary for function.

>>> We double-checked this work from Kesari et al 2012, they showed that intronic/noncoding natural variations are able to alter alternative splicing which in turn changes the amount of functional versus non-functional form of P5CS1 (which is in turn affecting proline content).

Line 401: this sentence is confusing. Please rewrite.

>>> This part about WUE has been removed to refocus the discussion according to your point #3)

Line 413: reciprocal planting experiments? Where? Statement is unclear. I think the authors mean a common garden across environments differing in temperature fluctuations and precipitations.

>>> We have modified this accordingly (now L339-341).

Fig. 4b: why so many points for Col-0/flm3? Are the 30 replicates shown here? Would a mean with error bars be more appropriate? The legend needs to be clarified.

>>> There are in total 128 data points consisting in 32 replicates per genotype: flm-3 and its wild type counterpart Col-0, as well as the lines rHIF99-Can and rHIF99-Col. The legend was changed accordingly.

>>>

Reviewer #2 (Remarks to the Author):

The middle of this paper (Results) describes a nice genetic dissection of some growth traits differences between 2 Arabidopsis lines. Hanemian et al. first grew RILs from the Can-0 and Col-0 cross and performed a QTL analysis. They then watched the major QTL segregate in the progeny of a residually heterozygous RIL, confirming the phenotypic effect. The QTL contained FLOWERING LOCUS M (FLM), so the authors went to nulls for this gene. Next, they performed a quantitative complementation assay in F1 hybrid plants to mutant flm-3. Finally, Hanemian et al. transformed the flm-3 mutant with the indicated sequence from both parental lines. In aggregate, these data show that allelic variation at FLM is responsible for growth and color differences between Can-0 and Col-0 owing to the QTL on chromosome 1. Subsequent work suggests that a SNP creates a splice site difference, causing the phenotype. In general, the work is a compelling demonstration of the power of forward genetics in

Arabidopsis.

The introduction needs a complete re-write. Despite much lingo and many broad statements, I could not tell what the paper was actually about until I read the results section (which is very clear). Regarding the motivation for the study, the fact that a mutation to a MADS-box transcription factor has effects on multiple phenotypes is not remotely surprising. Any mutation that affects growth or allocation will affect numerous plant dimensions through time. However, the fact that the Quantitative Trait Nucleotide (QTN) is a splice variant is notable discovery. While not unprecedented (line 359 onward), the result bears directly on the general question of what sorts of mutations make a QTN. This would be a better focus for the Introduction/Discussion.

>>> Thanks for your comments; however we think that our introduction needs to remain general as we start with studying widely integrative traits with the goal to better understand the relationships between traits. It is one of the 'beauty' of exploiting natural variation: to only discover the molecular mechanism of the variation once the QTN has been identified (if identified). Hence we find it difficult to introduce our story directly with splicing as we did not intend to screen for this type of variant. Still, the introduction has been shortened (see in the track change document between L92-118) and altered at several instances to avoid too general/long considerations, especially on pleiotropy. In addition, taking into consideration comments from the other reviewers, the discussion is now shortened and more focused on the relevance of our findings in terms of molecular variation and ecological/adaptive relevance (especially that of the LES).

>>

Reviewer #3 (Remarks to the Author):

The manuscript by Haneman et al describes how natural variation at the FLOWERING LOCUS M (FLM) gene contributes to pleiotropy in Arabidopsis. The authors mapped QTLs for several growth related traits, which turned out to be mapping to the FLM locus. When they analysed the FLM locus in strains that differed in the alleles, the underlying molecular mechanism hinted at changes in splicing. The authors go to length to try and establish that variation in splicing contributes to this variation across multiple growth related traits. The authors claim that Natural variation in FLM splicing mediates synergistic pleiotropy in Arabidopsis. The authors show that the alleles of interest have an interpretable geographic distribution indicative of adaptive significance. Overall the story is interesting, as it goes beyond flowering and it would be of interest to plant biologists and evolutionary biologists.
However the study suffers from few issues, which I have listed below, addressing which would substantially improve this manuscript.

This manuscript suffers severely from a chronological effect of experiments. In 2013/14, there was Nature paper published by Pose et al, which proposed a model based on an assumption that the primary transcripts arising from FLM are FLM-beta and FLM-delta. This assumption was incorrect and this mis-led the field and several researchers on a wrong track. This paper also appears to be one of those mis-led by this story and that is reflected in their interpretation.

> ***>>> The reviewer is correct about the chronological effect: when we started our work on FLM, the main paper about this gene was indeed from Pose et al 2013 and we started to study the transcripts of both FLM-beta and FLM-delta isoforms, while it was shown in further articles (Lutz et al 2015 & 2017; Sureshkumar et al 2016) that FLM-beta is the functional isoform, and that the change in splicing toward potentially non-functional isoforms is the mechanism in play. However, we think that our interpretation was right as it is close to what the reviewer writes below (see the paragraph starting with "FLM is a well-known…" L330 in our original version, and now L342 unchanged).***

When I look at the data they present it is very clear, what is really happening. In Col-0 you have primarily FLM-beta. In Can-0, you have a new splice acceptor site that is strong enough to outcompete the normal splice site that would have resulted in FLM-beta. Therefore, with the new splicing instead of AG1 (i.e., splice acceptor that is in front of Exon2) the new splice site is used resulting in a novel transcript, that contains a premature stop codon. The authors are too influenced by the beta/delta model presumably due to historical reasons, and fail to see this. As far as I could see their data clearly shows that the primary effect is loss of the normal (FLM-beta) transcript and formation of a novel transcript with a premature stop codon due to a hot splice acceptor site.

There is no need at all to discuss their work in the context of FLM-delta in this paper. This is the primary problem with this paper and it requires re-writing the paper with the current knowledge. The authors presumably are tip-toeing around this issue, which is not very helpful.

> ***>>> Although we think our interpretation was this one already, we agree that our Results section describing our work on other isoforms is not needed anymore regarding the current FLM model (hence we have deleted L198-211 in the Results of the previous version). Thanks to this reviewer's comment, this modification simplifies this section and avoids paying tribute to information which are now outdated.***

I have given some clear suggestions below.

Fig 1A:

rHIF068: The legend says it is fixed for FLM. The genotype panel indicates that orange is Col and Blue is Can. Then what do the colours indicate in rHIF68? Does this mean that rHIF068 that is fixed for Col and Can alleles for FLM do not differ in their phenotypes? Is this correct? Or rHIF68 is fixed for Col allele? This makes not much sense.

>>> The coloured boxplots indicate fixed alleles at the segregating region in each recombined-HIF (rHIF). Each rHIF is segregating for a fraction of the initial HIF region; we added in the legend that their genotype can be found in Table S4. rHIF068 is segregating for a region that does not include FLM (contrary to rHIF099) and, hence, does not differ in phenotypes. The legend has been improved for clarity.

Fig 1B & D.
16ºC panel figure.. Did all 21 plants flowered on day 30 in the flm mutant background? Is this what the figure shows? How was days to flowering measured. This lack of variation is a bit strange… I really have difficulties to believe that all 15 plants in Col flowered on day 54 and all 30 plants of flm flowered on day 47 or something. Please explain.

>>> Indeed, except for the 15 Col-0 plants of the Fig 1D, the plants did not flower all the same day. There was a mistake in the R script used to generate the boxplots where the outliers were removed by default. We corrected this (for all the figures containing a boxplot) and apologize for the confusion. An explanation of the homogeneity of the Col (and other) plants in terms of flowering time is the controlled conditions we grow them in (even on the Phenoscope robots during the vegetative period) and also the fact that we marked flowering only every other day (hence 'averaging' 2 days of flowering on a single date).

Fig 1C: What is this significance for? The data discussed in lines 181 and 183 is somewhat inconsistent with the figure. Please double check this analysis and its interpretation.

>>> The significance of the Fig1C corresponds to the pvalue (= 2.638e-05) mentioned line 181 (original version). We have added a sentence in the legend for clarity.

Lines: 194 to 195. Rephrase this.. This idea of discussing FLM splicing in the context of FLM beta and delta leads to fundamental confusion in the manuscript. It is better to state that FLM splicing/expression

>>> This has now been rephrased accordingly (see previous answers) .

Fig 2 and Fig S3. It think both these figures can be removed. They do not really add any value. It is pretty clear from this work as well as from earlier works that such analysis would lead to misleading conclusions. The authors have done a lot of work. But this does not help either the paper or the story itself. I suggest the authors to directly discuss the sequencing of full-length cDNA clones and remove this from the manuscript. The entire section of lines 194 to 213 can be removed. I believe all these discussions with exon2 vs exon 3 etc are not very useful. The authors can simply state that temperature affects FLM splicing and then present their cloning data. I suggest to make Fig S4 as the main fig S2, which can easily drive the point.

>>> Regarding the previous modification/deletion of the paragraph about FLM isoforms, we agree with the reviewer that the fig2 and FigS3 are not relevant anymore. We only kept the relative expression obtained by qRT-PCR with primers spanning the first intron as it is a good proxy for the loss of expression of FLM-beta in Can-0. This result now becomes the Fig2A. We then followed the suggestion of the reviewer by making the initial FigS4 the new Fig2B.

Lines 231 and 232: We have no idea whether the promotion of growth has got anything to do with these alleles. Should be rephrased to say that there was no differences in PRA were observed between plants harbouring either allele.

>>> What this shows is similar growth trajectories (and coherent with previously described growth phenotypes) of 2 different hypofunctional alleles at FLM, so we think our statement is cautious enough.

Lines 249-250: Unclear … what is being shown here and what does that mean?

>>> This is a statistical comparison of the Leaf Economics Spectrum (LES) trait covariations among different datasets (see the raison d'être of such analysis in the next response). To perform such a comparison, it is classical to test for potential differences in slopes among these datasets given that the slope reflects the strength of the physiological tradeoffs studied (see e.g., Reich et al. 2014 Journal of Ecology). The best practice to compare these slopes is to perform a Standard Major Axis (SMA) regression analysis because there is no a priori on the causal relationship between a trait X and a trait Y (compared to an OLS regression for instance). It has thus been argued to examine allometric lines with this technique (e.g. Warton et al 2012). This sentence thus gives the slope (issued from an SMA regression) for each dataset as well as the confidence interval (CI) of each slope. If there is no overlap of CI between two datasets, their slopes significantly differ.

Lines: 241-254: I have no idea what is meant by this. E.g., "variation observed across all plant species"???

>>> The LES (notably the relationship between Amass and LMA) was first identified in ecological interspecific studies, notably in Wright et al. 2004 (Nature) that analyzed the phenotypic variation of more than 2,000 species. The authors suggested that the tight relationship between Amass and LMA reflects a fundamental physiological constraints among plant species worldwide at the origin of their diversification and local adaptation (Reich et al. 2014 Journal of Ecology). However the adaptive significance of LES trait variation was not clearly demonstrated due to the lack of intraspecific data. More recently, Sartori et al (2019) made such a demonstration using hundreds of natural accessions of Arabidopsis thaliana. The LES should thus reflects leaf-level 'universal' physiological constraints at the origin of growth variation and local adaptation. It is thus important to test whether and how any population/genotype falls into this phenotypic spectrum to

identify its position along this universal spectrum. The LES is indeed seen both as a proxy for the photosynthetic machinery and as a physiological constraint from which it is difficult to evolve. As such, we aimed at comparing our data to the generic interspecific comparisons (data from Wright et al. 2004) and from the study analyzing the global LES variation in A. thaliana (data from Sartori et al. 2019). We have clarified this general objective in the text (now the 2nd paragraph of the discussion).

Lines: 285-300: Unclear. I am not sure whether this data is convincing enough.

>>> ***The section on relicts and admixture has been reviewed with the help of comments from reviewer #1.***

Lines 327-329: “which is expressed at a substantial level in many Arabidpsis strains”? where is this data from… I do not believe any paper has shown this clearly. I suggest to remove this.

>>> ***This has been removed.***

Lines 338: Remove in exon 3 etc.. A premature stop codon is a premature stop codon. End of story.

>>> ***Removed***

Lines 344-346 and the full line of argument: This is incorrect to state. Nd-1 and Ei-6 carries full deletion of the FLM locus. I am not sure how appropriate is the line of argument made in this paragraph.

>>> ***The reviewer is right. Indeed these 2 accessions were shown in Balasubramaniam et al 2006 to carry a full deletion at the FLM locus. Our statement that FLM is “preferentially targeted in non-coding region suggesting that a total loss of function might be too deleterious for the plant” was not adequate as well as the line of arguments of this paragraph. We changed it accordingly and focus on the type of mutation/QTN that may be selected in natural populations as suggested by reviewer2.***

Fig. S5B: Are there 2 Col-0 lanes?

>>> ***Yes, there are 2 Col-0 lanes.***

Reviewers' comments:

Reviewer #1 (Remarks to the Author):

I am examining the manuscript of Hanemian et al. for the second time. The manuscript has been greatly improved but I am still yet satisfied.

My main problem is that the findings presented in Fig 4 remain unclear. Does Fig. 4A show that flm has no effect on Amass (pvalue code is not indicated in the legend…)? What does Fig.4B show? That the covariance between LMA and Amass is not changed by FLM or that FLM has an effect on both LMA and Amass ? The term "trigger" is confusing, I don't think that it is the genotype that are the cause of the covariance between LMA and Amass, but rather physiological constraints, and these are also perceived in flm-3 and FLM-Can. In the text, the authors explain that the covariance between LMA and Amass is the same as in a worldwide sample of A. thaliana. If the slope does not change, and the effect is only significant on LMA, why do the authors conclude that FLM has a substantial effect on plant physiology? It appears to me that the effect of FLM is hardly detectable…
What is the data/analysis presented on line 225-227 and why do the authors compare its effect to the variance measured across 2206 species? Since environmental plasticity affects the measurements, what is the information that the authors draw from the comparison of their sample (grown in common garden set up) with the worldwide samples and species (see also line 309-310)? Based on their response to my question (rebuttal letter), it seems that this comparison should not be made.

Also, the authors do not seem to be very clear about the nature of the pleiotropic effect of FLM. Should the means of LMA and Amass change depending on FLM alleles or should it be the slope, in order for the authors to conclude that leaf physiology is changed by FLM? How to interpret the observation that an effect is detected in the HIF but not in the flm-3/Col-0 comparison?

I recognize that I am not an expert in the LES syndrome, and much of the readership of this paper will also not be. In my view (which I understand is also the author's view), the findings presented in Fig. 4 are central to the significance of the work. This figure and the corresponding paragraph must therefore be improved.

Note finally that the manuscript still does not really sort out the role of temperature. FLM function is temperature dependent (stronger effect at lower temperature) and involved in adaptation to temperature (not to temperature mean but to temperature variance). In addition, it impacts leaf temperature (the methods omits to explain how leaf temperature was measured). It is important to clarify all these aspects in the discussion. Are they functionally connected? Should one not expect that FLM variation would be more adaptive at low temperature (since its effect is stronger)? Is it not a contradiction that it is found to associate with temperature variance instead of mean? Does this have to do with mean leaf temperature?

Reviewer #2 (Remarks to the Author):

The authors made slight revisions to introduction.

[Editor: In the remark to Editor section, this reviewer specifically points out that the wordy Introduction problem was not addressed.]

Reviewer #3 (Remarks to the Author):

It appears to me most of my concerns have been appropriately addressed. The only minor suggestion

I would add would be to change the title to "Natural variation at FLM-splicing..." or "Splicing variation at FLM..." rather than "Splicing natural variation.." a double adjective term.

Hanemian et al.

Nature Communications

Point by point response to reviewers' comments

>>> Please refer to the track changes version of the text submitted to see the modifications in the text

Reviewer #1 (Remarks to the Author):

I am examining the manuscript of Hanemian et al. for the second time. The manuscript has been greatly improved but I am still yet satisfied.

My main problem is that the findings presented in Fig 4 remain unclear. Does Fig. 4A show that flm has no effect on Amass (pvalue code is not indicated in the legend…)?

1>>> In Fig4, we assessed the effect of variation at FLM on LMA and Amass traits individually, in 2 different genetic backgrounds. In the 4A right-hand side panel, when comparing the mutant flm-3 to its wild type counterpart (Col-0 genetic background), we indeed didn't detect a significant difference for Amass (although there seems to be a trend in the "expected" direction), while we noticed a significant difference using the rHIF099 genetic background. This is now described in more details in the text (L223-225 track changes version). As for the p-value code, we described the basic stats in the material and methods section (paragraph "statistical analysis") to avoid too busy legends.

What does Fig.4B show? That the covariance between LMA and Amass is not changed by FLM or that FLM has an effect on both LMA and Amass ?

2>>> In the Fig4B panel, we compare the covariance between LMA and Amass associated with the FLM function to the covariances observed at the intra- or interspecific level. As discussed in L294-297 (previously submitted version), LMA and Amass are two "core traits of the Leaf Economics Spectrum (LES), a universal trade-off between resource acquisition and resource conservation observed across thousands of plant species. The tight relationship between Amass and LMA is supposed to reflect fundamental physiological constraints among plant species worldwide, shaping their diversification and local adaptation".
So, we did not expect FLM to change the covariance between these 2 traits (if any) but rather to contribute to the variation observed along the LES by affecting multivariate phenotypes coordinately.
In other words, our answer is "yes" to both points raised here by the reviewer. FLM pleiotropically controls trait variation within a phenotypic space - the LES - that is highly constrained by physiological trade-offs and it has a subtle effect on both LMA and Amass as shown in Fig4A (although the effect on Amass is

less clear in the Col-0 genetic background as mentioned in the previous answer).

We want to mention here that we initially analyzed the covariance between LMA and Amass associated with variation at FLM in each genetic background separately. We observed a similar correlation in the Col-0 genetic background when using the flm-3 / WT comparison (standard major axis (SMA): $r2 = 0.21$, $P < 0.001$) and in the rHIF099 genetic background when using the rHIF099-Can / rHIF099-Col comparison (SMA: $r2 = 0.16$, $P < 0.01$). It is noteworthy that the correlation is stronger in the Col-0 genetic background although only LMA was significant when each trait was analysed individually (further explanation about SMA stats below). As the genetic backgrounds themselves were not associated with the covariance and were in similar phenotypic ranges, we decided to bulk all the data measured in this study in Fig4B (SMA: $r2 = 0.17$, $P < 0.001$).

The term "trigger" is confusing, I don't think that it is the genotype that are the cause of the covariance between LMA and Amass, but rather physiological constraints, and these are also perceived in flm-3 and FLM-Can.

3>>> We agree with the reviewer that "trigger" can be misleading as it suggests a link of direct causality between variables that we cannot demonstrate (discussed in L318-327, previously submitted version), and we thank the reviewer for pointing out this issue. We had indeed previously changed one instance of 'trigger' following this reviewer's suggestion but had overlooked three other instances, which are now modified as well into 'is associated with' (L225 & L345 track changes version and in the figure 4 legend). Then, if the covariance observed in our dataset was only caused by physiological constraints without genetic effect, we should not observe any difference between the pair of genotypes tested for each trait in the Fig4A panel. So our results show that the variations along the LES axis can be partly explained by allelic variations at FLM.

In the text, the authors explain that the covariance between LMA and Amass is the same as in a worldwide sample of A. thaliana. If the slope does not change, and the effect is only significant on LMA, why do the authors conclude that FLM has a substantial effect on plant physiology? It appears to me that the effect of FLM is hardly detectable…

4>>> Despite a slight and non-significant phenotypic variation observed for Amass due to flm-3 mutation, this variation was sufficient to recapitulate the LES correlation between LMA and Amass even when considering only the data of the Col-0 genetic background (see above first answer, SMA: $r2 = 0.21$, $P < 0.001$). For this, we used state of the art statistical methods (SMA) to calculate the correlation significance and compare the regression slopes of our FLM dataset with the A. thaliana natural accessions and the species datasets (obtained from literature). SMA tests are commonly used in trait-based ecology to measure regression slopes and test differences between groups (see for instance: Wright et al. Nature 2004; Price et al. PNAS 2007; Reich et al., PNAS 2014). In short, SMA takes into account the variation of both x and y traits,

while traditional ordinary least squares (OLS) regressions only minimizes the variation of the y-axis trait (for details see Warton et al. Methods Ecol Evol 2012); this explains why we recapitulate the LES correlation with the flm-3 mutation despite marginal variation for Amass. The SMA analysis also shows that the regression slopes are not significantly different from the one obtained using a worldwide sample of A. thaliana, thus suggesting that FLM functional variation is associated with changes in leaf physiology similarly to what is observed at the species scale.
Even if the effect of the variation at FLM on the LES continuum is not large (after all this is only the effect of a single gene's variation), it is remarkable that it is still quantifiable along the LES axes highlighting its contribution to the definition of plant's ecological strategies.
Overall, functional variation at FLM explains some of the variation in LMA and Amass, while remaining within the LES constraints. This is what we called physiological variation (along LES).

What is the data/analysis presented on line 225-227 and why do the authors compare its effect to the variance measured across 2206 species? Since environmental plasticity affects the measurements, what is the information that the authors draw from the comparison of their sample (grown in common garden set up) with the worldwide samples and species (see also line 309-310)? Based on their response to my question (rebuttal letter), it seems that this comparison should not be made.

5>>> Despite the confounding factor of plasticity shifting our phenotypic values away from those obtained in common garden experiments, the LMA/Amass correlation is very similar between flm datasets and others (especially intraspecific dataset);hence, we thought it was interesting to report the potential proportion of worldwide variation in LMA-Amass regression that could be caused by variation solely at FLM.
To avoid any overinterpretation of the relevance of this point we have now restricted this comparison of FLM effect to the Arabidopsis intraspecific dataset, especially because the regression slopes are directly comparable (although the intercept is not). We have also focused our comparison to the variation contributing to change along the LES spectra to be more robust against phenotypic plasticity. In other words, we now orthogonally project each point on the LES regression line, and compare the range of variation along the LES due to genetic variants at FLM with that found among Arabidopsis accessions. The overall figure is unchanged though (about 3%).

Also, the authors do not seem to be very clear about the nature of the pleiotropic effect of FLM. Should the means of LMA and Amass change depending on FLM alleles or should it be the slope, in order for the authors to conclude that leaf physiology is changed by FLM? How to interpret the observation that an effect is detected in the HIF but not in the flm-3/Col-0 comparison?

6>>> We showed that FLM alleles are associated with a change of LMA and Amass phenotypic values leading to a covariance between these traits that is very similar to the ones observed at the intra- of interspecific level. Thus, we

conclude that FLM has an effect on leaf physiology (not mentioning the leaf growth, color and temperature data). As we mentioned previously, we were not expecting FLM to change the universal LES relationship but rather to contribute to this relationship.

I recognize that I am not an expert in the LES syndrome, and much of the readership of this paper will also not be. In my view (which I understand is also the author's view), the findings presented in Fig. 4 are central to the significance of the work. This figure and the corresponding paragraph must therefore be improved.

7>>> The central significance of the work for us is that we have been able to isolate the phenotypic contribution (and potential adaptive and evolutionary significance) of a single polymorphism in a gene toward physiological variation, which happens to remain along the known LES.
We agree with the reviewer about the importance of clarity regarding the effect of FLM on LES-related traits and relationships. We modified and clarified the text accordingly (L220-253 & L330-360 track changes version).

Note finally that the manuscript still does not really sort out the role of temperature. FLM function is temperature dependent (stronger effect at lower temperature) and involved in adaptation to temperature (not to temperature mean but to temperature variance).
In addition, it impacts leaf temperature (the methods omits to explain how leaf temperature was measured). It is important to clarify all these aspects in the discussion. Are they functionally connected? Should one not expect that FLM variation would be more adaptive at low temperature (since its effect is stronger)? Is it not a contradiction that it is found to associate with temperature variance instead of mean? Does this have to do with mean leaf temperature?

8**>>> The reviewer points out that the 3 different types of data we have related to temperature are not enough discussed together. We did not do so as it is not always possible to draw straightforward connexions between them (although it may be interesting).**
1- FLM is indeed a repressor of flowering whose active form is down-regulated when temperature increases. We used this characteristic to validate FLM as a good candidate gene underlying our QTL of interest in Fig 1B. Then, in further experiments, a temperature between 16 and 21°C (i.e. a temperature at which the FLM repression still occurs) was used in order to highlight functional differences between the allelic forms of FLM.
2- We established a significant correlation between the polymorphism affecting FLM function in the arabidopsis accessions of the Iberian Peninsula and the temperature heterogeneity occuring in nature at the place where these accessions were collected (Fig 5C), suggesting an adaptation to this environmental variable.
3- We showed that FLM is controlling leaf temperature (Sup. Fig 5) as a proxy for transpiration state. We apologize for the oversight regarding the leaf temperature in the method section and thank the reviewer for having noticed it. This now appears in Materials & Methods (L489-491 track changes version).

Regarding point 3 (leaf temperature), we included it in the study because we thought it would be of interest in the context of the pleiotropic role of FLM on leaf physiology. However, we cannot make a straightforward link between the temperature of the leaf and point 1 and/or point 2.

As reviewer1, we also thought it was noteworthy that variation at FLM among natural accessions was correlated with temperature heterogeneity, knowing that FLM is a gene whose function is temperature-dependent. That's why we elaborated about that in the discussion (L375-382, previously submitted version). But as this part of the discussion is speculative, we did not go much further in this direction. For instance, temperature fluctuations in the data set we used range from -8°C to 36°C, while most of the experiments on FLM in the literature were performed in controlled conditions with temperature between 15°C and 27°C (without day or night fluctuation). Moreover, we don't know so much about how the temperature signal is integrated by FLM in a natural environment. Does the temperature need to be constantly above a certain threshold to be translated in a visible phenotype or is it enough to go above the threshold once/regularly? So far, we know that 8-12 hours at 27°C -under otherwise stable and controlled conditions- is a sufficient period of time to change the splicing profile of FLM in plants initially grown at 16°C (Sureshkumar et al 2016). Overall we think these are interesting questions that might be addressed in another study.

Reviewer #2 (Remarks to the Author):

The authors made slight revisions to introduction.

[Editor: In the remark to Editor section, this reviewer specifically points out that the wordy Introduction problem was not addressed.]

Reviewer #3 (Remarks to the Author):

It appears to me most of my concerns have been appropriately addressed. The only minor suggestion I would add would be to change the title to "Natural variation at FLM-splicing..." or "Splicing variation at FLM..." rather than "Splicing natural variation.." a double adjective term.

>>> Thank you for your comment, the title is now : "Natural variation at *FLM* splicing has pleiotropic effects modulating ecological strategies in *Arabidopsis thaliana*"

Reviewer #1 (Remarks to the Author):

I thank the reviewers for their illuminating explanations. I am fine with the correction made to the manuscript.
